# Psychologically informed oral health interventions in pregnancy and type 2 diabetes: A scoping review

**DOI:** 10.3389/froh.2022.1068905

**Published:** 2022-12-21

**Authors:** Camilla Böhme Kristensen, Mark Ide, Angus Forbes, Koula Asimakopoulou

**Affiliations:** ^1^ Centre for Host-Microbiome Interactions, Faculty of Dentistry, Oral & Craniofacial Sciences, King's College London, London, United Kingdom; ^2^ Care in Long Term Conditions, Florence Nightingale Faculty of Nursing, Midwifery & Palliative Care, King's College London, London, United Kingdom

**Keywords:** oral health, pregnancy, diabetes, behaviour change, oral health promotion, gestational diabetes mellitus

## Abstract

**Introduction:**

Oral health is a critical aspect of gestational diabetes management. Gestational diabetes is high blood glucose levels during pregnancy and is managed like type 2 diabetes with diet and physical activity interventions. This scoping review sets out to discuss why oral health support should also become part of gestational diabetes management.

**Objectives:**

The primary objective was to synthesise the existing psychologically informed oral health interventions for pregnant women and individuals with type 2 diabetes, and the extent to which these interventions map on to the COM-B Model. No literature exists on oral health interventions in gestational diabetes, why studies with type 2 diabetes populations were selected instead. The secondary objective was to identify the precise outcomes targeted in the interventions.

**Methodology:**

The Joanna Briggs Institute's Methodology for Scoping Reviews was used to conduct this review. The populations of interest were pregnant women and individuals with type 2 diabetes, and eligible concepts were psychologically informed oral health interventions. Quasi-experimental and experimental designs were considered. The Ovid Interface including Embase, Medline, Global Health, APA PsychInfo, Health Management Information, Maternity, Infant Care Database, the Cochrane Library, and CINAHL was used as information sources. The study selection followed the PRISMA guidelines. The first search was conducted on the week commencing the 25th of July 2022, with a follow-up search conducted on the 10th of October 2022.

**Results:**

28 records were included for synthesis. The most frequently assessed psychological outcome was oral health knowledge and the most frequently assessed oral clinical outcome was Plaque Index. All studies used an educational intervention approach, while psychological capability in the COM-B Model was targeted in all interventions by increasing oral health knowledge among the participants. The Health Belief Model was the most frequently used theory in the interventions.

**Conclusion:**

The results demonstrate that oral health is a recognised aspect of pregnancy and type 2 diabetes. The findings from this review and a qualitative interview study which is under development will inform the first oral health intervention for women with gestational diabetes in the United Kingdom.

## 1. Introduction

A detailed discussion of the literature is found in the published study protocol (1), hence, a summary of the literature on oral health, gestational diabetes, and pregnancy is presented in this review. Periodontal disease is a common oral health complaint that affects around 45% of the adult British population (2). Periodontal disease is an umbrella term for gingivitis which is inflammation of the gums and periodontitis which is an advanced disease with the destruction of the bone and tooth-supporting and periodontal tissue (2). Periodontal disease can cause loose teeth and tooth loss, pocket formation in the gums, bad breath, and receding gums which can lead to nutritional deficiencies because of poor food uptake (3). In addition to physical consequences, it is also associated with poor quality of life (4). The treatment of periodontal disease is a collaboration between the dental team and the patient (5).

Gestational diabetes is high levels of blood glucose that first occurs in pregnancy and affects around 15% of the global population. It is the cause of 80% of pregnancy-related complications. The blood glucose levels return to normal after delivery. Lifestyle interventions with diet and physical activity modifications are used to manage gestational diabetes. Although this is a temporary condition, it is associated with risks of hypertension, hemorrhage, and increased risks of type 2 diabetes later in life for the mother. Babies born to mothers with gestational diabetes have increased risks of large birth weight which can cause complications during delivery, low blood glucose levels at birth, and a life-long increased risks of obesity and type 2 diabetes (6).

Oral health and periodontal disease have multi-directional relationships with diabetes (7–11), including gestational diabetes (12, 13). Firstly, one study reported that women with gestational diabetes had a higher prevalence of periodontal disease compared to women with normoglycemic pregnancies (12). Meta-analysis has further shown that baseline periodontal disease is a risk factor for gestational diabetes development (13), highlighting the two-way relationship.

Moreover, poor oral health and periodontal disease is independently associated with low birth weight and premature birth (14), while normoglycemic pregnancies are associated with compromised oral health with around 40% of women showing clinical signs of gingivitis (15). However, as the criteria to assess periodontal disease varies, the significance of the relationship between adverse pregnancies and periodontal disease depends on the clinical assessment methods used (16). Nevertheless, the relationship between oral health and pregnancy outcomes is very well established.

The relationship between periodontal disease and adverse pregnancy outcomes is described in detail in the published protocol (1). Briefly, the systemic link between the two variables is thought to be due to the inflammatory responses of the host (17). This hypothesis has had support from animal studies where induced periodontal disease was associated with adverse pregnancy outcomes in hamsters (18, 19). In humans, we know that successful periodontal therapy that improves the periodontal status can improve cardiovascular markers (11, 20).

The inflammatory hypothesis is also implicated in the bi-directional relationship between periodontal disease and diabetes (21, 22). Bacteria from periodontal disease in the oral cavity may enter the circulation and cause inflammation and insulin resistance leading to raised blood glucose levels (22). On the other hand, research shows that individuals with diabetes have larger amounts of advanced glycation end-products in their oral cavity compared to non-diabetes individuals, which can lead to an inflammatory response causing damage to the periodontal structures (22).

The maintenance of good oral health is dependent on the patients' daily oral hygiene practices and treatment help seeking behaviours (23). It is the healthcare professional's responsibility to advise patients on their oral health and provide oral health treatments. However, given the high prevalence of periodontal disease globally (2), it is evident that the general population's oral hygiene practices and treatment help seeking behaviours are suboptimal. While it is recommended to brush teeth twice daily, a recent survey suggested that 29% of British people brushed their teeth once per day, while 2% stated that they don't brush at all (2).

Fortunately, there is extensive evidence to suggest that behavioural interventions can be effective in inducing oral health behaviours. Behavioural interventions based on theoretical modelling of behaviour are more effective compared to behavioural interventions that are non-theory driven (24). However, extensive behavioural theories exist, and it has been a problem for behavioural scientists to select the most appropriate theories, citing the many available frameworks which are often overlapping and have interrelated constructs and components (25). To overcome this issue, researchers have attempted to collate the most common constructs of the available behavioural theories in to one model, which has resulted in the Capability-Opportunity-Motivation-Behaviour Model (COM-B Model) and associated Behaviour Change Wheel which is a framework for intervention design (26).

The COM-B Model proposes that a given behaviour will occur when an individual has the *capability* and *opportunit*y to engage with the *behaviour,* and when the individual is *motivated* to enact the specific behaviour (over any other behaviours). The three components are furthermore divided in to psychological and physical capability, social and physical opportunity, and reflective and automatic motivation. Capability and opportunity are influencing the relationship between motivation and behaviour, instead of the behaviour itself. Consequently, these components need to be available for motivation to generate the behaviour. Therefore, a highly capable individual, or an individual with the belief that they can perform a behaviour, and the more conducive the environment is to enact a behaviour, the greater likelihood of a behaviour to occur. The behaviour also feeds back to all three components (capability, opportunity, and motivation), creating either a positive or negative feedback cycle (27). For example, when enacting a behaviour that requires skill, practicing or rehearsing the behaviour will improve capability that will increase the motivation to continually engage in a behaviour. Conversely, if an individual experience failure in performing a behaviour, or if the environment does not encourage a behaviour, this may decrease the individual's belief that he/she is not capable of performing the behaviour (28).

In the context of oral health, it is evident that the COM-B Model framework is used increasingly to guide oral health interventions. Buchanan, and colleagues (2020) conducted a systematic review to identify oral and dental interventions that had used these frameworks and a total of nine studies fulfilling the inclusion criteria were identified (25). Using frameworks when developing behavioural interventions provide standardization which allows for replication of the intervention in other populations (28).

## 2. Rationale

As demonstrated, the literature suggests that periodontal disease, gestational diabetes, and pregnancy are interrelated. Firstly, pregnant women experience increased risks of poor oral health (15), while periodontal disease is associated with adverse pregnancy outcomes (14). Periodontal disease and diabetes, including gestational diabetes, are further interrelated, highlighting why oral health support should form part of gestational diabetes management. Psychological interventions modelled on theory is a useful method for inducing new behaviours, including oral health behaviours (25).

### 2.1. Objectives

Therefore, the primary objective was to describe the existing psychologically informed oral health interventions for pregnant women and individuals with type 2 diabetes, and the extent to which the interventions map on to the COM-B Model. The secondary objective was to identify the precise outcomes targeted. A psychologically informed intervention is defined as an intervention that targets psychological and/or behavioural outcomes. An intervention can still be psychological if a psychological theory is not used to guide the intervention development, providing the outcomes targeted relate to psychological constructs such as knowledge and behaviour (29).

The results from this review and a qualitative study which is in progress are used to inform the development of a new oral health intervention for women with gestational diabetes. This is the first attempt (to our knowledge) in the United Kingdom to promote oral health in this population using behavioural science.

## 3. Methodology

The authors conducted the scoping review in accordance with the Joanna Briggs Institute Methodology for Scoping Reviews (30).

### 3.1. Protocol deviations

The protocol is published in BMJ Open (1). There were minor deviations from the protocol. The Allied and Complimentary Medicine (1985–2022) and the CINAHL databases were included as additional information sources as they were deemed relevant. The protocol stated that participants should be over 18 years of age; however, an initial search identified several studies including pregnant women of any age, citing the importance of targeting oral health in women considered at high risk (i.e., underaged). As periodontal disease affects∼40% of women of reproductive age (31), it was decided to disregard the minimum age requirement as initially stated in the protocol.

### 3.2. Eligibility criteria

In accordance with the Joanna Briggs Institute methodology (30), the participants, concept (intervention and outcome), context, and study design were used to guide the inclusion and exclusion criteria (Supplementary Table S1). The eligibility criteria are described in detail in the published protocol (1), hence a summary is provided below.

#### 3.2.1. Eligible participants

Eligible studies included participants who were pregnant women at any age and any gestational age, and studies which had included participants with type 2 diabetes. Participants from all socio-economic classes and ethnicities were considered as these are relevant factors in oral health promotion (32).

#### 3.2.2. Ineligible participants

Studies with non-pregnant participants and individuals with type 1 diabetes were excluded. Gestational diabetes is associated with a later risk of type 2 diabetes and is managed like type 2 diabetes (33), why studies with type 1 diabetes participants were deemed ineligible.

#### 3.2.3. Eligible and ineligible concepts

Eligible study concepts included psychologically informed oral health interventions that were designed to target oral health-related behaviours. As discussed previously, psychologically informed oral health interventions relate to interventions that target psychological and/or behavioural outcomes such as knowledge and behaviour (29). Ineligible concepts were studies where interventions were periodontal therapy only, and where no outcomes were psychologically related (e.g., behaviour).

#### 3.2.4. Context

All contexts were considered for this study.

#### 3.2.5. Study designs

Study designs including quasi-experimental and experimental designs and systematic reviews where the research questions were relevant to this review were deemed eligible. Mixed method studies were eligible if there was a clear separate reporting of the quantitative and qualitative data. All other study designs were excluded.

### 3.3. Search strategy and information sources

The search strategies found in Supplementary Appendix A for the Ovid Interface and in Supplementary Appendix B for CINAHL were developed by reviewing the search strategies of relevant systematic reviews from the Cochrane Library. The search strategy for the Cochrane Database is in Supplementary Appendix C. A librarian from King's College London provided feedback on the search strategies and changes were made. The search terms were derived from four categories: *oral health, intervention, pregnancy, and type 2 diabetes*. Studies published in Danish and English were considered. The initial search was conducted on the week of the 25th of July, with a follow-up search conducted on the 10th of October 2022 prior to submitting this paper.

The Ovid Interface (2022) was used to access the following databases: EMBASE + EMBASE Classic (1974 to 2022), Ovid MEDLINE(R) (1946 to 2022), Global Health (1973 to 2021), APA PsychInfo (1,806 to 2022), HMIC Health Management Information (1979 to 2021), Social Policy and Practice and Maternity and Infant Care Database (1971 to 2022). The Cochrane Library, the EU Clinical Trials Register (https://www.clinicaltrialsregister.eu/) and the OpenGrey database was sought for randomised controlled trials, and grey and/or unpublished literature respectively. The CINAHL database was also sought.

### 3.4. Data management and study selection

Covidence (www.covidence.org) was used to manage the data. The study selection followed the PRISMA (Preferred Reporting Items for Systematic Reviews with Meta-analysis) process with *identification*, *screening* (title and abstract), *eligibility* (full text screening), and *inclusion* (34).

### 3.5. Data extraction, analysis, and presentation

Three data extraction tools were developed and/or used to address the research objectives of this review. Firstly, a data extraction tool extracting information relating to the study characteristics including first author details, year, country, study population characteristics, study design, sample size and follow-up, main findings and outcomes were developed. Secondly, the Template for Intervention Description and Replication (TIDieR) Checklist by (35) was used to describe the interventions. This checklist extracted information relating to the first author details and year, the why (rationale), what (materials and procedures), who provided (intervention facilitators), how (mode of delivery), where (setting), when and how much, tailoring and fidelity. Lastly, a data extraction tool was developed to extract information about the psychological theories used in the interventions and how the interventions map onto the COM-B Model. The results were presented in text and tables using narrative synthesis.

## 4. Results

**Figure 1:**
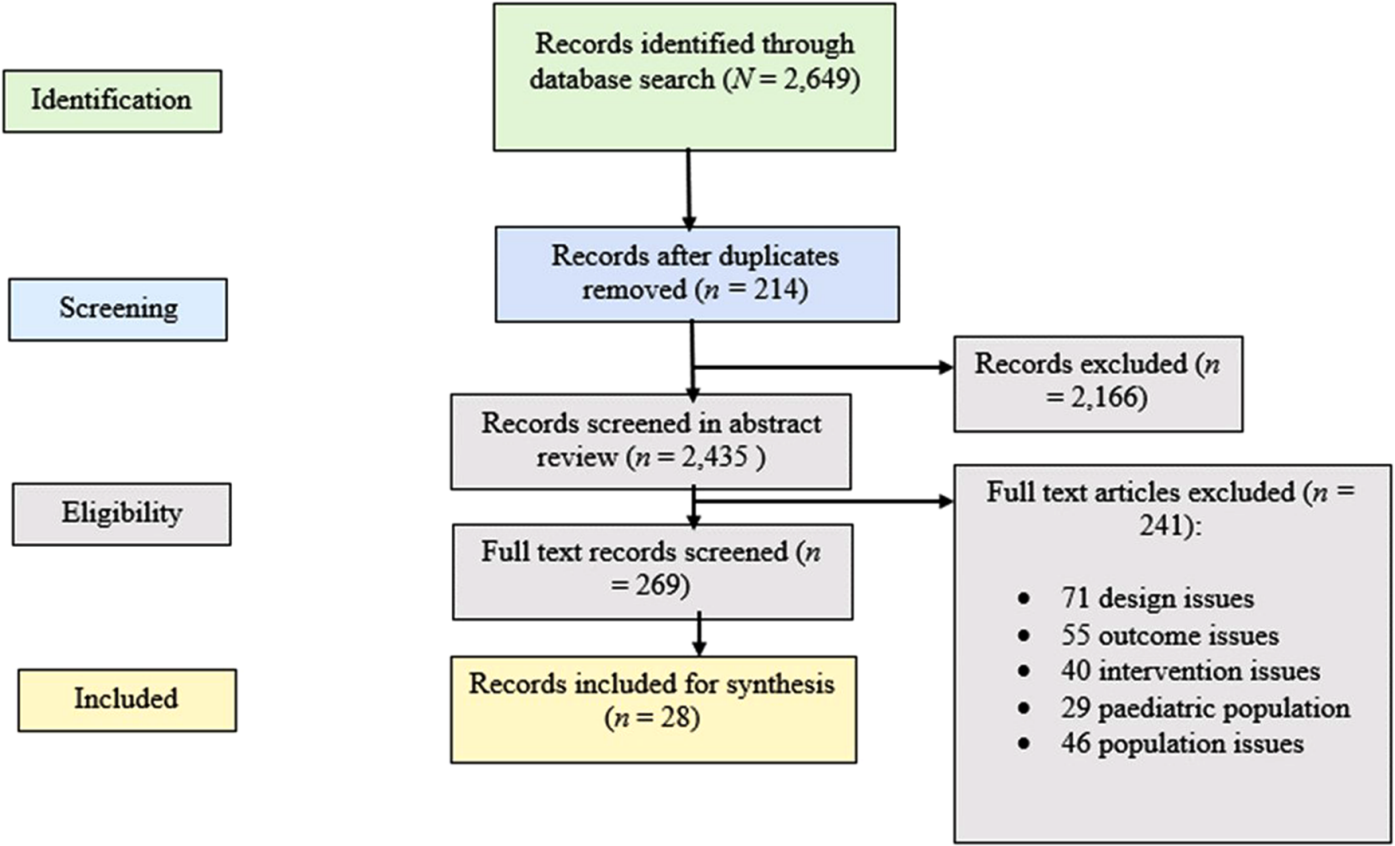
Details the number of records identified, included and excluded, and the reasons for exclusions.

The search identified 2,649 records. After removing duplicates, 2,435 records were screened to assess eligibility using the title and abstract. 2,166 records were excluded as they were not relevant, leaving 269 records that were examined in full text. Of these, 241 records were excluded in the full text screening because of ineligible study designs, outcomes, intervention types and populations. This process resulted in 28 records being included for synthesis, with 20 studies conducted with pregnant women and seven conducted with patients with type 2 diabetes. Figure 1 details the exclusion reasons and how many records were excluded for each criterion.

Supplementary Tables S2A,B describe the study characteristics, including the first author details, year and country, population characteristics, study design, sample size and follow-up, the main findings, and outcomes for pregnant women (Supplementary Table S2A) and patients with type 2 diabetes (Supplementary Table S2B). Supplementary Tables S3A,B describe the interventions using the TIDieR Checklist (35), where data relating to the study aim, materials, procedure, intervention facilitator, mode of delivery, intervention setting, frequency of intervention delivery, details on intervention tailoring, and intervention fidelity are summarised. The results from the studies with pregnant women are summarised in Supplementary Table S3A, while the studies with type 2 diabetes patients are summarised in Supplementary Table S3B. Supplementary Tables S4A,B detail how the included interventions map onto the COM-B Model. The studies with pregnant women are detailed in Supplementary Table S4A, while the studies with type 2 diabetes patients are detailed in Supplementary Table S4B.

### Study settings, population characteristics, designs, and outcomes

Most studies were conducted in Iran (10 studies), followed by the United States (5 studies), India (3 studies), Australia (2 studies), Thailand (2 studies), Turkey (1 study), Kuwait (1 study), Taiwan (1 study), Denmark (1 study), and the United Kingdom (2 studies). The studies with pregnant women had various ethnicities including East-Asian (Thailand), Southeast Asian (India), and Middle Eastern (Iran) participants, while the studies conducted in the United States recruited women of African American (90% of the sample) (36), Hispanic (70% of the sample) (37), and White ethnicities (79% of the sample) (38). The eight studies with type 2 diabetes patients had some ethnic diversity with participants from Turkey, Denmark, Taiwan, Iran, and Thailand. The lowest participant age was reportedly 16 years in the pregnant population and ranged from 30 to 70 years in the type 2 diabetes population. Seventeen studies reported on educational achievement. Four studies with pregnant women but, surprisingly, none with type 2 diabetes patients reported on ethnicity. Eleven studies reported on the socio-economic status by stating monthly or yearly income, or by stating what socio-economic class the participants belonged to. The socio-economic reporting was assessed using self-report. Only two studies reported on co-morbidities by means of self-report.

The most used study design was randomised controlled trials (13 studies), followed by quasi-experimental designs (7 studies), pre-test-post-tests (4 studies), and systematic reviews (3 studies). The sample sizes ranged from 40 to 639 participants, and the longest follow-up was 18 months. The main findings of the studies are summarised in Supplementary Tables S2A,B. Thirteen studies evaluated outcomes relating to oral clinical health by assessing variables such as Plaque Index, sites bleeding on probing, Gingival Index, decayed, missing, and filled teeth and Clinical Attachment Levels. All 28 studies evaluated psychological and behavioural outcomes by assessing a range of variables such as oral health knowledge, oral health attitudes, self-efficacy, toothbrushing and flossing behaviour, perceived barriers and benefits and susceptibility. Fourteen studies evaluated both oral clinical health outcomes and psychological and/or behavioural outcomes. Oral health knowledge improved in (94%) of all studies at follow-up in the intervention arms.

It appears that global efforts are being made to promote oral health in pregnant women and individuals with type 2 diabetes, citing the different geographical locations of the studies. Most studies considered socio-economic status as important, and this was self-reported across studies using education, income, or socio-economic class. There appears to be a trend for clinical impact on oral health status across studies, with ten studies finding that clinical oral health markers improved post-intervention.

### Interventions

All 28 interventions were focused on oral health education in pregnancy or type 2 diabetes. The materials are described in detail in Supplementary Tables S3A,B, but consisted of resources such as PowerPoint slides, provision of leaflets, oral health toolkits, booklets, and audiovidual aids. The intervention procedures, as well as details on ‘where’, ‘when and how much’ are described in Supplementary Tables S3A,B. All 28 studies included interventions delivered face to face, and most were group-based. Two studies with type 2 diabetes patients and one study with pregnant women delivered the intervention over the phone in conjunction to face to face. Different personnel such as nurses, midwives, dentists, health coaches, counsellors, and study researchers delivered the interventions. Ten studies tailored the intervention to the individual participant by providing individualised counselling, lifestyle and dietary advice. Seven studies assessed fidelity and detailed how the intervention facilitators ensured adherence to the intervention protocol. Examples of fidelity assessment included a standardised script that the intervention facilitators should follow (39), while another study assessed fidelity by reviewing audiotapes of the intervention facilitators delivering the intervention (37).

The results showed that all studies used an educational intervention approach where the study participants were educated, using in-person mode delivery, about oral health in pregnancy and diabetes, respectively. Nurses and oral health professionals were most frequently facilitating the interventions.

### Theory and intervention mapping using the COM-B model

The Health Belief Model (8 studies), was the most commonly used theory underpinning the interventions, followed by Motivational Interviewing (6 studies). Other theories including self-efficacy theory, Social Cognitive Theory, Theory of Planned Behaviour and Neurolinguistic Programming were also used. Ten studies did not report a psychological or behavioural theory for their intervention. The psychological capability domain in the COM-B Model was targeted in all 28 interventions by increasing knowledge about the importance of oral health in pregnancy or type 2 diabetes. Psychological capability also relates to an individual's ability to comprehend information about a behaviour. The physical capability domain in the COM-B Model relates to the individual's physical skills in performing a behaviour. This was targeted in four interventions by providing training to the participants on oral hygiene behaviours. The physical opportunity domain in the COM-B Model relates to environmental restructuring that provides an individual opportunity to engage with the target behaviour. This domain was targeted in 14 interventions by providing the participants with oral health toolkits containing toothbrushes, dental floss and toothpaste, or by offering free dental health appointments. The social opportunity domain in the COM-B Model was not addressed in any interventions. Social opportunity relates to opportunities as a result of social factors such as social cues or cultural norms (27). The motivation domain in the COM-B Model was targeted the least across studies. Reflective motivation was targeted in three studies by encouraging the participants to plan their oral health-related behaviours, or by implementing behavioural self-monitoring. Reflective motivation entails conscious efforts to plan out a behaviour.

## 5. Discussion

This scoping review synthesised the existing psychologically informed oral health interventions for pregnant women and individuals with type 2 diabetes, and the extent to which these interventions map on to the COM-B Model. This review also identified the precise outcomes targeted in the interventions.

Vamos and colleagues (2015) conducted a systematic review on oral health promotion interventions in pregnant women where seven studies were identified. The authors noted that there remained a significant gap in oral health promotional efforts for pregnant women. The results of this scoping review identified 20 oral health promotion intervention studies designed for pregnant women, suggesting increased attention to promoting oral health in pregnancy. This is encouraging citing the evidence suggesting that∼40% of pregnant women demonstrate clinical signs of periodontal disease (gingivitis) (15).

In addition, this review also identified eight studies promoting oral health in type 2 diabetes which is favourable, citing the epidemiological evidence suggesting bi-directional relationships between poor oral health and diabetes. Furthermore, the National Institute for Health and Care Excellence's recent guidelines recognised that individuals with diabetes are at increased risk of periodontitis and that efforts to manage periodontitis in people with type 2 diabetes should be made to improve blood glucose control.

### Population characteristics

The identified oral health interventions were conducted in several countries with different types of ethnicities. Periodontal disease is associated with ethnic disparities (40), hence, the inclusion of ethnically diverse samples is important in oral health promotion. Not all studies reported on educational achievements or monthly or yearly income. Education and income are indicators of socio-economic status, which is relevant in periodontal disease prevalence (41). Moreover, education and income may impact what is considered healthy, normal or acceptable oral health by a participant. Therefore, better reporting of relevant socio-economic factors among the participants in the sample is needed in future studies aiming to promote oral health.

The non-reporting of co-morbidities was an issue across studies. Co-morbidities are important in oral health, as evidence suggests that periodontal disease is associated with an increased risk of 29% of acute myocardial infection after adjusting for confounding factors such as diabetes and smoking socio-economic factors (42). Likewise, other evidence suggests that there is a bi-directional relationship between chronic kidney disease and periodontal disease as demonstrated in a study using data from over 11.000 adults (43). Better reporting of co-morbidities in studies investigating the effectiveness of an oral health intervention is therefore needed to account for relevant confounding factors.

### Study designs

While the randomised controlled trial designs are considered the most reliable evidence to assess the effectiveness of an intervention (44), quasi-experimental designs pose limitations to the study's ability to conclude a causal association between the oral health intervention and desired outcomes. Therefore, the effectiveness of the interventions with non-randomised methodologies should be interpreted with caution, and issues relating to for example low validity due to differences in characteristics between the intervention and control group participants should be considered when making conclusions about the intervention effectiveness (45). The follow-up period across the studies ranged from four weeks to 36 months. A longer follow-up period is more desirable in oral health interventions, as oral health-related behaviour change should be long-term rather than short-term (46). Although oral health-related behaviour may only be particularly critical during pregnancy citing the evidence suggesting changes in the oral health status during this time period; long-term oral health behaviours are important in preventing oral disease across the lifespan (47).

### Outcomes

The studies which assessed Plaque Index all found a statistically significant improvement in participants who received the intervention across study designs, and a trend towards clinical impact was observed. Plaque Index has been demonstrated as a reliable and reproducible marker of clinical oral health (48). Psychologically informed oral health interventions may therefore be as relevant in improving clinical markers of oral health as interventions with periodontal therapy only. Oral health knowledge and other self-reported psychological outcomes were assessed differently across studies. While some studies constructed ‘their own’ oral health knowledge questionnaires based on oral health facts derived from a literature review (49), or by validation of experts in the field (50); other studies assessed oral health knowledge using theoretically derived questionnaires (39, 51). The different assessments of self-reported psychological or behavioural outcomes pose an issue to the generalisability of the effectiveness of the interventions due to different signalling questions, directions, and units (52). It is therefore only possible to look at the intervention effectiveness within studies, rather than across studies on self-reported outcomes. The development of oral health-related psychological measures will be an important contribution to research methods in this area as previously noted by Renz and colleagues (46).

Around half of the studies assessed Plaque Index in conjunction with self-reported psychological or behavioural outcomes. However, the relationship between clinically determined and self-reported oral health behaviour is complex, and there is a general discrepancy between self-reported oral health and periodontal disease (53, 54). To overcome this issue, a calibrated statistical model of clinical and self-reported oral health was developed by Liu and colleagues (2010). This model suggested that general health conditions, the number of times a person has received healthcare, as well as gender, age, education, and income should be included as relevant factors that will moderate the discrepancy between clinical and self-report oral health. Future studies that aim to assess the effect of an oral health promotion intervention may wish to use this calibrated model. Furthermore, there is often a need to validate self-reported oral health against formal clinical assessment because of the discrepancy between self-reported oral health and periodontal disease (55, 56).

### Interventions

The studies generally described their rationale for conducting the study, the materials used, the procedures, how the intervention was delivered (e.g., face to face), where it was delivered and ‘how much’ was delivered. However, eight studies did not state who facilitated the intervention. Furthermore, around half of the studies did not tailor the intervention to the participants which is of concern considering the evidence suggesting that tailoring may enhance the intervention impact (57). Tailoring of an intervention refers to ‘*any combination of information or change strategies that are intended to reach one specific person, based on characteristics that are unique to that person, related to the outcome of interest and have been derived from an individual assessment’* (58).

### Theory and the COM-B model

The Health Belief Model was the most frequently used model in the identified interventions in this review and has been widely used in other health behaviour promotion interventions (55). However, some studies suggest that only some of the Health Belief Model components (perceived susceptibility, severity, and benefits) are relevant domains in oral health-related behaviour (56, 59). Nevertheless, it is encouraging to see behaviour change theory being utilised in intervention studies, citing the evidence suggesting that theory-based interventions are more effective than non-theory-driven interventions in changing behaviour (60).

While all interventions targeted psychological capability by means of educating the participants about oral health; research has shown that education is a passive form of intervening and that more strategies are needed to induce behaviour change (27). For example, targeting psychological capability with oral health education about the importance of brushing and flossing teeth is not enough if an individual does not have the physical skills (such as manual derexity) to engage with correct brushing/flossing techniques. Likewise, targeting psychological capability with oral health education about the importance of brushing/flossing is not enough if an individual lacks the physical opportunity (i.e., oral health appliances) to perform the behaviour. For example, the cost of purchasing floss may be a physical barrier to engaging with recommended oral hygiene behaviours. It has, therefore, been suggested that all three domains of the COM-B Model should be considered within the oral health setting to induce behaviour change (61).

Automatic motivation and social opportunity were not targeted in any interventions. According to the COM-B Model and associated Behaviour Change Wheel framework which can be used to design interventions; automatic motivation is best targeted by regulation (establishing rules of behaviour or practice), or legislation (making or changing laws), or by service provision (e.g., by providing free dental service). This may explain why automatic motivation was not targeted in any of the interventions, as these were focused on individual participant behaviour, as opposed to community-wide initiatives such as regulating behavioural practice. Likewise, according to the Behaviour Change Wheel, the social opportunity may best be targeted by environmental/social planning which refers to designing and/or controlling the physical or social environment (27). This implies that this COM-B domain may be easier to target in community-wide interventions rather than person-specific interventions. Moreover, as previously noted, not all identified interventions were based on theory providing an opportunity for the oversight of important influential factors on behaviour change (62). Research further suggests that interventions that claim to be based on theory often are not (63), leading to the oversight of important intervention functions that can address relevant determinants of behaviour.

### Limitations

The findings of this review should be considered in light of the noted limitations. Firstly, this review is unable to generalise the findings about the intervention effectiveness, as each study used different scales and methods to assess oral health-related psychological outcomes. Therefore, only conclusions about the individual study's effectiveness can be drawn here. Secondly, it may be that only studies with statistically significant results published their research, leading to publication bias (64, 65). Thirdly, quality assessment of the included studies does not form part of usual scoping review methodology; hence the inclusion of studies with poor quality may have occurred. Ongoing discussions on the need for quality assessment in the scoping review methodology continue. However, this scoping review was conducted in accordance with current guidelines and therefore no quality appraisal was completed (66).

## Conclusion

The findings from this review demonstrate that oral health is becoming a recognised aspect in pregnancy and type 2 diabetes, citing the increase in studies aiming to promote oral health in these populations over the recent years. Most interventions were focused on oral health education using face to face delivery. There was a trend towards clinical impact on Plaque Index and oral health knowledge across studies. The Health Belief Model was the most frequently used theory across studies, while psychological capability in the COM-B Model by means of increasing knowledge about oral health was targeted in all studies. Several studies did not consider important influences on behaviour such as social influence or motivation.

### Strengths and implications

This review is the first to synthesise the psychologically informed oral health interventions designed for pregnant women and individuals with type 2 diabetes. It is encouraging to see that oral health promotional efforts are being made for pregnant women and individuals with diabetes, citing the evidence highlighting the importance of oral health in these populations. However, oral health interventions for women with gestational diabetes are still missing, despite the evidence suggesting that oral health is a critical aspect of positive health outcomes for these women and their fetus. Women with gestational diabetes report feeling like ‘baby making machines’ and often experience highly medicalised pregnancies (67). They experience issues specific to gestational diabetes that ‘regular’ pregnant women and individuals with diabetes may not encounter. It is therefore critical that efforts are being made to develop an oral health intervention tailored specifically for women with gestational diabetes that address their specific needs and experience.

The findings from this review and a qualitative interview study with women with gestational diabetes (in progress) will be used to inform the development of the first (to the authors’ knowledge) oral health intervention for women with gestational diabetes in the United Kingdom. Based on this review, it appears that an intervention with an oral health educational component targeting oral clinical outcomes and psychological outcomes such as oral health knowledge may be a good starting point. However, as several of the identified interventions in this review overlooked some of the influential domains (e.g., automatic motivation and social opportunity) of behaviour, it is important that these are considered in a novel oral health intervention for women with gestational diabetes to ensure effectiveness and long-term behaviour change.

## Data availability statement

The original contributions presented in the study are included in the article/**Supplementary Material**, further inquiries can be directed to the corresponding author/s.

## Author contributions

CBK has contributed to the planning and conduct of the abstract, introduction, methodology, results, and discussion. Professor MI has contributed to the introduction section regarding periodontal health, AF has contributed to the introduction section concerning the diabetes literature, and KA has contributed to the introduction section surrounding psychological approaches in oral health. All the above-mentioned persons have given their permission to be mentioned in this manuscript. All authors contributed to the article and approved the submitted version.

## Funding

This study is part of CBK's Doctoral thesis funded by the Faculty of Dentistry, Oral & Craniofacial Sciences at King's College London from October 2021 to August 2025.
