# Supplementary material for: Psychologically informed oral health interventions in pregnancy and type 2 diabetes: A scoping review
Source: Front Oral Health. 2022 Dec 21;3:1068905. doi: 10.3389/froh.2022.1068905 (PMC9811123; doi:10.3389/froh.2022.1068905)
Supplement: Supplementary file 1 [file Table1.docx]

**Supplementary File**

**Table 1**

*Inclusion and exclusion criteria for study selection derived from the participants, concepts (intervention and outcome), context (setting), and study design.*

|  | Inclusion | Exclusion |
| --- | --- | --- |
| Participants | - Pregnant women of any gestational age - Individuals with type 2 diabetes - Women with gestational diabetes | - Individuals with type 1 diabetes or other types of diabetes |
| Concepts (intervention) | - Psychologically^1^ informed oral health interventions | - Interventions with periodontal therapy only where no psychologically informed component is present |
| Concepts (outcome) | - Psychological, behavioural, and clinical oral health-related, pregnancy and diabetes-related outcomes such as: - Oral health behaviour (e.g., toothbrushing) - Oral health knowledge, awareness, and importance - Oral health clinical markers (e.g., Plaque Index) - Diabetes markers (e.g., HbA1c) - Pregnancy outcomes (e.g., weeks at birth) - Self-efficacy - Studies with both psychological and oral clinical markers were eligible | - Studies where oral clinical markers were not reported in conjunction with psychological outcomes were ineligible |
| Context (setting) | - All settings and contexts | - Not applicable |
| Study design | - Randomised controlled trials - Pre-test post-test studies - Quasi-experimental studies - Systematic reviews - Mixed-method studies where there’s a clear reporting of the quantitative data | - Observational studies - Qualitative studies - Non-experimental studies |

**Table 2a**

*Study characteristics of studies with pregnant women.*

| First author, year, country | Population characteristics | Design | Sample size *N* | Follow-up | Main findings | Outcomes |
| --- | --- | --- | --- | --- | --- | --- |
| Adams, 2017, USA(1) | Pregnant women over 18 years of age. The average age was 28 years. Most of the sample was Hispanic (70 %). Most women had achieved above High School qualifications (45 %) for the intervention arm and (55 %) for the control arm. (53 %) and (58 %) in the intervention and control arm respectively, had Public Health Insurance. (57 %) and (61 %) in the intervention and control arm had attended a dentist <1 year ago. The income was >$20000 per year for (54 %) of the sample | Quasi-experimental pilot study | 101 | 9 weeks post intervention | Statistically significant improvements were observed in Plaque Index (*p* = 0.01), bleeding on probing (*p* = 0.01), and pocket depths (4 mm or greater), (*p* = 0.01) in the intervention arm only from pre-test to post-test. There was a small improvement in oral health importance in the intervention arm compared to the control arm, (*p* = 0.05). No differences were seen in oral health knowledge, self-efficacy, and toothbrushing/flossing frequency | - Plaque Index - Sites bleeding on probing - Pocket depths - Oral health knowledge - Oral health importance - Oral health self-efficacy - Toothbrushing/flossing frequency |
| Al Khamis, 2017, Kuwait(2) | Pregnant women over 18 years in the second trimester. The mean age in the intervention arms were 28 and 27 years and 26 years in the control arm. (14 %, 10 % and 7 %) of the women across the arms had a High School degree. (10 %) of the women were housewives/not working. Most women reported having a monthly income of <£750 per month. The average number of children were two per participant | RCT with three arms | 154 | 4 weeks post intervention | The Plaque and Gingival Index improved across intervention arms and was statistically significant. No statistically significant differences were observed for toothbrushing/flossing frequency. All women across intervention arms improved their oral health knowledge, oral health attitude, and social norms to oral health and perceived fewer barriers to brushing/flossing and snacking. Perceived barriers to dental attendance did not change in any arms | - Plaque Index - Gingival Index - Oral health knowledge - Oral health attitude - Subjective norm - Toothbrushing barriers - Flossing barriers - Snack barriers - Barriers to dental attendance (NS) - Toothbrushing/flossing frequency (NS) |
| Bahri, 2015, Iran(3) | Pregnant women with a gestational age between 12 and 28 weeks. The mean age was 25 years in the intervention arm and 24 years in the control arm. Most women were housewives (90 % in both arms) and had achieved a Diploma qualification (66 % in the intervention arm and 73 % in the control arm). Gingivitis was present in (49 %) of the intervention arm participants and in (36 %) of the control arm participants | RCT with two arms | 160 | 2 months post intervention | The oral health beliefs and toothbrushing/flossing frequency were significantly higher in the experimental arm immediately post-intervention and at two-months follow-up, (*p* = 0.05) | - Oral health beliefs - Toothbrushing/flossing frequency |
| Bansal, 2019, India(4) | Pregnant women in the first and second trimester. The mean age was 29 years and ranged from 18 to 42 years. (28 %) had High School qualifications and (30 %) had a Postgraduate qualification. (67 %) of the women were non-working homemakers. (37 %) identified as upper-middle class, while (35 %) identified as lower-middle class | Pre-test post-test | 200 |  | The oral health knowledge and oral health attitude scores significantly improved from pre-test to post-test, (*p* = 0.01). Pre-scores were found to be associated with the level of education (*p* = 0.01), and socioeconomic class (*p* = 0.01). The change in the post-score from pre-score was statistically significant in all the categories of age, education, occupation, and socioeconomic class, (*p* = 0.01). There was a statistically significant difference in the post-knowledge scores among the pregnant women belonging to different socioeconomic classes, (*p* = 0.01) | - Decayed, missing, and filled teeth - Gingivitis - Toothbrushing frequency - Oral health knowledge - Oral health attitude |
| Cardenas, 2010, USA(5) | Pregnant women between 21 and 38 years of age with a mean age of 26 years. The women were between the 12^th^ and 40^th^ weeks gestation. All women were English speaking | Pre-test post-test | 40 | 1 month post intervention | All women improved their oral health knowledge from pre to post-test. The knowledge scores went from 12.9 (with 53 % correct answers) to 20.9 in the post testing (with 87 % correct answers), (*p* = 0.01). The questions which most women answered wrong in the pre-test related to the effects of alcohol intake during pregnancy, pacifier use, emergency management of dental trauma, transmission of bacteria and formation of dental caries in children. In the post-test none of these questions were answered wrong or missed more than 50 % of the time | - Oral health knowledge |
| Chawla, 2017, India(6) | Pregnant women with a mean age of 26 years. 40 were from a high socio-economic class, 36 were from the middle socio-economic class and 36 were from the low socio-economic class. There was a relationship between healthy periodontal tissue and socio-economic class | Pre-test-post-test | 112 | 7 months post intervention | After the intervention, knowledge regarding oral health care improved significantly. Attitudes toward oral health became more positive, but oral health practices did not change. Periodontal health post intervention improved more in women from a high socio-economic class | - Oral health knowledge - Oral health attitudes - Oral health practices - Community Periodontal index - Decayed, missing, and filled teeth - Prosthetic status |
| Cibulka, 2011, USA(7) | English speaking pregnant women, with most women (39 %) in the intervention and control arms aged between 21-24 years. The women were pre-24-weeks in their pregnancy. Most women were African American (90 %) in the intervention and control (72 %) and were single. Around half of the women in both arms had achieved High School qualifications. Most women and in the intervention arm (82 %) and control arm (93 %) had Medicaid (public health insurance). Most women (68 %) had a yearly income of <$10000 | RCT with two arms | 170 | At 36-weeks pregnant | There were no statistically significant differences in oral health knowledge between the arms. There was a significant increase in (better) subjective oral health and toothbrushing/flossing frequency in the intervention arm participants only. Dental visits in the last year increased in the experimental arm (*p* = 0.01) only. The women in the intervention arm ingested less sugary drinks at post-test compared to the women in the control arm | - Subjective oral health - Tooth brushing/flossing frequency - Oral health knowledge (NS) - Dental visits in the last year - Intake of sugary drinks |
| Clifford, 2012, Australia(8) | Pregnant women (~30 weeks gestation) over 18 years of age recruited from pre-natal clinics. The mean age was 28 years. (42 %) in the intervention arm were first time mothers, and the parity ranged from 1 to 9 children. Compared to the local population, the sample was more highly educated and less likely to be a single parent. Most women across the arms (81 %) identified as Australian | Quasi-experimental, longitudinal study | 611 | 4 months post birth | There was no statistical difference between the arms in tooth brushing frequency and dental visits. Four months after the birth of their infant, relative to the usual care condition, each of the oral health education interventions had independent or combined positive impacts on the mother’s knowledge of oral health practices | - Toothbrushing frequency (NS) - Dental visits in the last two years (NS) - Oral health knowledge |
| Ebrahimipour, 2016, Iran(9) | Pregnant women aged between 16 and 40 years old. Most women were between 26 and 30 years old and had achieved a college or university degree. Most women had a monthly income of <$300 per month and did not have a job. There was no statistically significant difference in the demographic characteristics between the intervention and control arm at baseline | Quasi-experimental study with 2 arms | 150 | 2 months post intervention | At follow-up, there were statistically significant improvements in the experimental arm only on the Theory of Planned Behaviour constructs outlined in the next column, (*p* <0.05) | - Oral health knowledge - Oral health attitude - Subjective norms - Perceived behavioural control - Intentions - Oral health performance |
| George, 2018, Australia(10) | Pregnant women between 12- and 20-weeks’ gestation recruited from maternity hospitals. The mean age in all arms were 29 years. Most women (72 %) were English speaking and (54 %) had achieved tertiary education. Around half (49 %) were from the lowest socioeconomic class and (56 %) were unemployed. (35 %) had public health insurance. There was no statistical difference in the baseline characteristics between the arms | RCT with three arms | 639 |  | Statistically significant improvements were observed in dental visits, oral health knowledge, quality of oral health, and oral health outcomes (bleeding on probing, dental plaque, Clinical Attachment Loss, decayed and filled teeth) in participants allocated to the Midwife intervention arm. No statistical differences were found between the arms in birth outcomes | - Dental visits - Oral health knowledge - Quality of oral health - Bleeding on probing - CAL - Plaque Index - Decayed teeth - Filled teeth - Birth weight (NS) - Gestational age at birth (NS) |
| Ghaffari, 2018, Iran(11) | Pregnant women in the first trimester of pregnancy with no serious dental health issues. The mean age in the intervention arm was 27 years and 26 years in the control arm. Most women in the intervention arm (78 %) and control arm (82 %) had been married for <10 years and (57 %) and (61 %) of the women in the intervention and control arm, respectively were housewives | RCT with two arms | 135 | 2 months post intervention | There was a statistically significant increase in oral health awareness, perceived susceptibility, perceived severity, benefits, barriers, self-efficacy, and toothbrushing/flossing frequency in the intervention arm only, immediately post-intervention. Two months later, there was a statistically significant difference in all outcomes in the intervention arm only, except for perceived susceptibility | - Oral health awareness - Perceived severity - Perceived benefits at follow-up - Perceived barriers at follow-up - Self-efficacy - Toothbrushing/flossing frequency - Perceived susceptibility (NS) |
| Jeihooni, 2017, Iran(12) | Pregnant women without progressive oral disease and a mean gestational age of 19 weeks were recruited from health centres. The mean age of the intervention arm was 28 years and 27 years in the control arm. Most of the women had health insurance (92 %) and were unemployed (72 %) | RCT with two arms | 110 | 4 months post intervention | There was a statistically significant increase in oral health knowledge, perceived susceptibility, severity, benefits, barriers, self-efficacy, and toothbrushing/flossing frequency in the intervention arm only at follow up. No statistically significant difference was seen in internal cues to action | - Oral health knowledge - Perceived susceptibility - Perceived severity - Perceived benefits - Perceived barriers - Self-efficacy - Toothbrushing/flossing frequency - Internal cues to action (NS) |
| Ramazani, 2014, Iran(13) | Pregnant women were recruited from a health centre. The mean age was 23 years. (77 %) of women in the ‘direct’ arm had a Diploma degree, while (26 %) and (56 %) in the ‘indirect’ and control group had Diploma degrees. Most women in the ‘direct’ arm were in the third trimester, while the women in the remaining groups were in the second trimester | Quasi-experimental study with three arms | 90 | 2 months post intervention | At follow-up there was a statistically significant difference in oral health knowledge, oral health behaviour and Modified Gingival Index (MGI) between the three arms (*p* = 0.05). No statistically significant differences were observed in oral health attitude between the groups. The ‘direct’ intervention arm method was shown to be the superior method in increasing pregnant women’s oral health knowledge and behaviours | - Oral health knowledge - Attitude (NS) - Oral health behaviour - MGI |
| RenuRawat, 2021, UK (14) | Pregnant women | Systematic review | Various | Various | The relevant studies from the systematic review have been described. See:   - Chawla et al., 2017 - Ebrahimipour et al., 2016 - Ramazani et al., 2014 |  |
| Riedy, 2015, USA (15) | English-speaking pregnant women in the first or second trimester of pregnancy. The mean age was 24 years. Most women where White (79 %) and around half (48 %) were primiparous. (60 %) had High School or less and over half (60 %) were married. (24 %) had been to the dentist in the last six months, (27 %) had been 1-2 years ago and (33 %) had been >2 years ago. The sample was predominantly White (79 %) and (8 %) were Hispanic | RCT with four arms | 400 | 18 months post-partum | There were no statistically significant differences between the Motivational Interviewing and health education arms for the mother’s pre-natal attendance (*RR* = 0.98, 95 % CI: 0.93 – 1.04), or for the children’s attendance in the remaining two comparison groups: post-natal Motivational Interviewing versus health educations (*RR* = 1.03, 95 % CI: 0.82 – 1.28), or pre-and post-natal Motivational Interviewing versus pre-natal health education/post-natal Motivational Interview arms (*RR* = 1.05, 95 % CI: 0.80 – 1.36). There were no statistical differences in preventive oral health practices of mothers in the Motivational Interviewing and health education arms and no difference between the pre-natal/post-natal Motivational Interviewing arm and the health pre-natal health education and post-natal Motivational Interviewing arm | - Prenatal dental attendance (mothers) (NS) - Postnatal dental attendance (child) (NS) - Pre- and post-natal toothbrushing/flossing frequency (NS) |
| Saffari, 2020, Iran (16) | Pregnant women recruited from health clinics. (60 %) were <30 years and most women (57 %) were >10 weeks pregnant. (57 %) had a university degree and (73 %) were housewives. Most women had 0-2 children (85 %) and perceived their oral health to be good (51 %), fair (40 %) and bad (9 %). Most women (92 %) had no co-morbidities. (50 %) of the sample reported a ‘moderate’ income | RCT with two arms | 112 | 3 months post intervention | A statistically significant improvement in general self-efficacy, oral health self-efficacy, toothbrushing/flossing frequency, Gingival Index (*p* = 0.01), and decayed teeth, (*p* = 0.05) were observed in the experimental arm only | - General self-efficacy - Oral health self-efficacy - Toothbrushing/flossing frequency - Gingival Index - Decayed teeth |
| Selvarajan, 2017, India (17) | Pregnant women between 22 and 35 years of age from a low socio-economic status | Pre-test-post-test | 138 | 3 weeks post intervention | There was a statistically significant increase in knowledge of nutrition and dental health (72 % at pre-test to 96 % at post-test), knowledge on oral health (69 % at pre-test to 98 % at post-test), and knowledge of preventive strategies for oral disease (65 % at pre-test to 97 % at post-test). There was a statistically significant increase in attitudes on oral health (73 % at pre-test to 99 % at post-test), and attitude toward oral hygiene (94 % at pre-test to 99 % at post-test) | - Oral health knowledge - Oral health attitudes |
| Shanazi, 2016, Iran (18) | Primiparous women in the first trimester of pregnancy. The mean age was 24 years. (40 %) of the sample had achieved High School, and (56 %) considered their income as ‘moderate’ | Quasi-experimental | 88 | 2- and 4-months post intervention | There was a statistically significant increase in oral health knowledge, perceived susceptibility, and self-efficacy at both follow-ups in the intervention arm only. No statistically significant improvements were seen in decayed, missing and filled teeth (DMFT) from pre- to post-intervention in the experimental arm when compared to the control arm | - Oral health knowledge - Susceptibility - Self-efficacy - DMFT (NS) |
| Nickbinposhtamsary, 2018, Iran (19) | First-time pregnant women with oral/tooth disease were recruited. Most women (84 %) were <30 years of age. (45 %) had High School qualifications. (67 %) were between 14-28 weeks pregnant. (65 %) of the sample reported an income of 5-10 million Rial | RCT with two arms | 110 | 3 months post intervention | A statistically significant improvement in oral health knowledge, perceived susceptibility, severity, benefits, barriers, and toothbrushing/flossing frequency were observed in the intervention arm only at follow-up. Plaque Index improved significantly in the intervention arm only at follow-up | - Oral health knowledge - Susceptibility - Severity - Benefits - Barriers - Toothbrushing/flossing frequency - Plaque Index |
| Vamos, 2015, USA (20) | Pregnant women | Systematic review | Various | Various | The relevant studies from the systematic review have been described. See:   - Cilbulka et al., 2009 - Cardenas et al., 2010 |  |
|  |  |  |  |  |  |  |

| Reference, country | Population characteristics | Design | Sample size *N* | Follow-up | Main findings | Outcomes |
| --- | --- | --- | --- | --- | --- | --- |
| Cinar, 2014, Turkey (21) | Individuals with type 2 diabetes (T2D) aged between 30 and 65 years of age, without psychological issues. The average time of diagnosis was 13 years and 10 years for the intervention and control arm, respectively | RCT with two arms | 186 | 6 months post intervention | At post intervention, the experimental arm had statistically significant lower HbA1c, better toothbrushing self-efficacy and more stress reduction compared to the control arm. There were statistically significant reductions in Clinical Attachment Levels (CAL) in the intervention arm. Participants in the intervention arm maintained their bodyfat levels while the participants in the control arm increased their bodyfat levels. Both arms improved diabetes self-management | - Toothbrushing self- efficacy - Diabetes distress - HbA1c - CAL - Bodyfat levels - Diabetes self-management |
| Cinar, 2018, Turkey/Denmark (22) | Danish and Turkish individuals with T2D aged between 30-70 years old. Participants had no severe psychological and somatic illness. Most individual in the Turkish sample were 50-59 years old (55 %) and most in the Danish sample were >60 years old (56 %). (54 %) in the Turkish sample had achieved Primary School or less, and (31 %) in the Danish sample had achieved Primary School up to 10^th^ grade | RCT with two arms | 288 | 6 months post intervention | The post-intervention results showed a statistically significant reduction of HbA1c (*p* = 0.01), Community Plaque Index (CPI), and an increase in toothbrushing frequency in the intervention arm participants only | - HbA1c - (Turkish sample) CPI - (Danish sample) CPI - Toothbrushing frequency |
| Hsu, 2021, Taiwan(23) | Patients with T2D aged 35 to 65 years with severe periodontal status. The mean age was 54 years. (51 %) were male and the mean BMI was 27 (classified as overweight). (48 %) had achieved a High School degree. (14 %) were smokers, and (23 %) considered their oral health as very good/good, (57 %) common, and very poor/poor (20 %) | RCT with two arms | 76 | 1-, 3-, and 6-months post intervention | The intervention arm participants showed greater improvement in oral health knowledge, and oral health attitude, pocket depth, and Clinical Attachment Level (CAL) than the control arm. Oral health-related quality of life (QoL) increased from first to last follow-up in both groups. The intervention arm participants increased their toothbrushing/flossing frequency compared to the control arm. No statistical difference in HbA1c levels were observed | - Oral health knowledge - Attitude - Tooth brushing frequency - CAL - Gingival Index - Plaque Index - Bleeding on probing - Oral health QoL - HbA1c |
| Malekmahmoodi, 2020, Iran(24) | Individuals with T2D and no oral symptoms were recruited. The mean age was 53 years, and most were females (66 %) and had health insurance (98 %) and were married (96 %). Most had completed Elementary School (71 %) in the intervention arm and 58 %) in the control arm. (85 %) of the sample reported a ‘moderate’ income level | RCT with two arms | 120 | 3-month post intervention | A statistically significant improvement was seen in the intervention arm only for oral health knowledge, perceived susceptibility, severity, barriers, benefits, self-efficacy, internal cues to action, and toothbrushing/flossing frequency. No improvements were seen for external cues to action | - Oral health knowledge - Susceptibility - Severity - Barriers - Benefits - Self-efficacy - Internal cues - Toothbrushing/flossing frequency |
| Phetnin, 2020, Thailand(25) | Older patients with uncontrolled T2D and chronic periodontitis were recruited. The mean age was 65 in the intervention arm and 67 in the control arm. Most participants were female in the intervention (80 %) and control (71 %) arm. The average time for T2D diagnosis was 12 years. Hypertension was the most common co-morbidity in the intervention (34 %) and control (28 %) arm. Most participants across the arms had never smoked. Most participants had finished Primary School (94 % in the intervention arm and 83 % in the control arm) | RCT with two arms | 70 | 3- and 6-months post intervention | All the Health Belief Model constructs (perceived susceptibility, severity, benefit, barriers, and self-efficacy) improved significantly in the intervention arm only at the last follow-up. There was a statistically significant difference in HbA1c and oral hygiene status (OHS) between the arms at follow up | - Susceptibility - Severity - Benefits - Barriers - Self-efficacy - HbA1c - OHS |
| Saengtipbovorn, 2015, Thailand(26) | Elderly patients with T2D | Quasi-experimental study | 113 | 3- and 6-month post intervention | At follow-up participants in the intervention arm had statistically significant higher knowledge and attitude towards oral health and T2D. The participants in the intervention arm were more likely to exercise, modify diet, have foot examinations, always wear covered shoes, participate in self-feet screening, use dental floss, and use inter-proximal brushes compared to the control arm | - Oral health knowledge - Oral health attitude - Diabetes attitude - Exercise frequency - Adherence to diet - Attend foot examinations - Toothbrushing/flossing frequency |
| Saengtipbovorn, 2014, Thailand(27) | Elderly patients with T2D who were >60 years of age. The mean age in the intervention arm was 63 years and 64 years in the control arm. Most were female (65 %) and between 60 to 69 years old (85 %). Most individuals had completed Primary School in the intervention (80 %) and control (72 %) arm. The average time for diagnosis was 8 years in the intervention arm and 6 years in the control arm. (83 %) in the intervention arm and (89 %) in the control arm had health insurance | Quasi-experimental study | 132 | 3-month post intervention | A statistically significant improvement was observed in the intervention arm participants only in HbA1c, fasting plasma glucose, oral health markers, oral health knowledge, diabetes knowledge, and attitude towards oral health and diabetes | - HbA1c - Fasting plasma glucose - BMI (NS) - Plaque Index - Gingival Index - Pocket depth - CAL - Oral health knowledge - Diabetes knowledge - Oral health attitude - Diabetes attitude |
| Ucheka, 2021(28) | Patients with T2D | Systematic review | Various | Various | The relevant studies from the systematic review have been described. See:   - Cinar, 2014 - Cinar, 2018 - Saengtipbovorn, 2015 |  |

**Table 2b**

*Study characteristics of studies with individuals with type 2 diabetes.*

**Table 3a**

*Intervention description of the studies with pregnant women using the TIDieR Checklist*

| Reference | Why (rationale) | What (materials) | What (procedures) | Who provided | How | Where | When and how much | Tailoring (individual) | Fidelity |  |
| --- | --- | --- | --- | --- | --- | --- | --- | --- | --- | --- |
| Adams, 2017 | To examine a skills-building oral health education intervention for pregnant women implemented in an existing maternal care program | The Eastman Toothpick Test (self-administered) was given to the intervention arm to test for signs of gum inflammation  Toothbrushes, fluoride toothpaste, dental floss and a timer were provided to the women in the intervention arm to enable opportunities to brush and floss at home  Leaflet with instructions on how to brush and floss were provided to the women as part of the educational material in the intervention arm | The CenteringPregnancy is a group care model often found in clinics with low-income populations in USA. It focuses on healthcare, interactive learning, and community building. Around ten women of similar gestational age attend each group (10 x 2-hour long sessions). The women began each session by recording clinical, pregnancy-related markers such as weight. This is followed by group discussions and interactive activities addressing relevant pregnancy health topics.  The oral health intervention was implemented and delivered at the third and fourth session in the programme described above. The topics of the sessions included importance of maternal oral health, common oral health problems and the safety and importance of dental care during pregnancy. Two activities: proper toothbrushing techniques and the Eastman Toothpick Test were provided.  The control group received treatment as usual (TAU), i.e., the regular CenteringPregnancy program without the dental health education | Nurse-midwives delivered the intervention and underwent a three-hour training on intervention content and delivery  The intervention was developed by dentists, periodontists, 1 x behavioural scientist, and facilitators from the existing maternal programme | Face to face (group) | Four San Francisco pre-natal care sites | 10 x 2-hour sessions (existing maternal care program)  2 x 2-hour oral health sessions were implemented at the third and fourth existing sessions | No | Yes, audiotapes to evaluate fidelity | |
| Al Khamis, 2017 | To examine if dental health education with or without planning increased the frequency of pregnant women’s’ oral hygiene behaviours | Oral hygiene leaflets were given to all arms. It contained information about brushing and flossing techniques  Toothbrush, family sized toothpaste and dental floss were provided to all arms  Dental assessment (Plaque Index and Gingival Index)  Standardised scripted explanation of the information in the leaflet were developed to ensure intervention fidelity  Culturally appropriate booklets about oral health and pregnancy were given to the women in the dental health education arm and the dental health+planning arm. The booklet targeted social cognitions including knowledge, attitudes, subjective norms, barriers, and intensions. The booklet was designed based on evidence from a previous qualitative study which was conducted by the researchers | There were three arms; the control group who received an oral hygiene leaflet and oral health supplies, the dental health education arm who received oral health education from a booklet that targeted social cognitive constructs, and oral health supplies, and the dental health+planning arm who received the same dental health education (booklet) described above, oral health supplies plus instructions to write down a plan of when, where and how they would brush and floss their teeth, what obstacles would hinder oral health behaviours and how to overcome obstacles. The intervention was delivered individually in a private office. The three arms received the same interaction time | Study researchers facilitated the intervention  1 x hygienist recruited the women and performed the dental assessment | Face to face (individual) | Three governmental maternity outpatient clinics in Kuwait | 1x session delivered in a private office | No | Yes, standardised script to ensure adherence | |
| Bahri, 2015 | To examine the effects of an oral and dental health educational intervention on pregnant women’s beliefs and behaviours on oral and dental health | Oral health education material (unspecified) was used to deliver the educational intervention | The intervention arm received an oral health education programme designed to address oral health issues in pregnancy  The control group received no education/TAU | Not mentioned | Face to face (group) | Health clinic | 6 x sessions over three weeks | No | No | |
| Bansal, 2019 | To examine oral health status, oral hygiene practices, and self-perception about oral health among pregnant women, and to assess the effectiveness of an oral health education on the oral health knowledge and attitudes of pregnant women | Oral health booklet developed on evidence-based guidelines were given as educational material. It was pilot tested and subsequent changes were made were needed. The booklet provided education about common dental diseases such as caries, healthy oral habits during pregnancy and infant oral care methods  Oral health pamphlets were given to the women following the intervention to reinforce the imparted learning  Dental assessment (dental caries, presence/absence of gingival bleeding, dental trauma, dental erosions, and oral mucosal lesions) | The intervention consisted of oral health education which was conducted face to face using the materials described | 1 x public health dentist conducted the dental health assessment  No mention of who facilitated the oral health education | Face to face (individual) | Maternity outpatient ante-natal clinic at a tertiary care hospital | 1x session of 20 minutes | No | No | |
| Cardenas, 2010 | To examine the gain in oral health knowledge after education in pregnant women on dental anticipatory guidance and to determine how much oral health-related information pregnant women retain over time | Oral health educational material (PowerPoint presentation) formed part of the educational delivery | The intervention consisted of a ten-minute presentation on oral health education. Pre and post tests were conducted at visit one, while a follow-up test was scheduled for visit two one month later | Not mentioned | Face to face (group) | Pre-natal clinic | 1 x session for 10-minutes | No | No | |
| Chawla, 2017 | To examine the effect of oral health education on pregnant women’s knowledge, attitude, practice, oral health status and treatment needs | PowerPoint slides with oral health information were used to deliver the education. It was focused on the prevention of dental caries and periodontitis through dietary and oral hygiene counselling | The women received oral health education using the material described and blanket dental health referrals | Not mentioned | Face to face (unclear if individual or group) | Hospital | Not described | No | Training was given to the oral examiners, but no measures were in place to ensure adherence to the intervention protocol | |
| Cibulka, 2011 | To test the effectiveness of an advanced practice nurse model of care to improve oral health in low-income pregnant women | Toothbrush, fluoride toothpaste and dental floss was provided to the intervention arm at the time of enrolment. The control group received these items after completion of the study  A DVD about oral health and proper brushing and flossing techniques was used as part of the educational material in the intervention arm  Graphic pictures about oral health formed part of the educational material in the intervention arm  Information sheet on oral health was used as part of the educational material for the intervention arm  Postcards were sent as reminders for the oral health check-up to women in the intervention arm | The intervention arm received an educational intervention that consisted of watching a 5-minute DVD about oral health. After, a nurse or researcher discussed the information on the information sheet and gave the women the oral health supplies. An appointment for an oral health check-up was scheduled for each woman. Reminder postcards were sent to the women 1 to 2 weeks prior to the scheduled appointment. The control group was TAU | Study researchers and nurses | Face to face (group) | Pre-natal clinic | 1 x session with oral health education  1 x session for oral health check-up | Yes, individual oral health check-ups and treatment plan (if needed) | No | |
| Clifford, 2012 | To examine the impact of oral health education provided to pregnant women on subsequent practices within the infants’ family | ‘Healthy teeth for life’ video (nine minutes long) was loaned to the women.  A bag containing a toothbrush, toothpaste, a ‘Zero to six’ oral health pamphlet developed by Colgate, a Johnson and Johnson ‘Teeth and teething’ pamphlet, and a fridge magnet which contact details to the local health service | The women in the first intervention arm labelled ‘video intervention’ were provided with the ‘Healthy teeth for life’ video described under materials which could be watched at home or in clinic. No measures were in place to check if the women had watched the video at home.  The women allocated to the ‘bag arm’ received the dental supplies described in the materials and were also invited to watch (at home or in clinic) the ‘Healthy teeth for life’ video in clinic.  The ‘combination’ arm was offered both resources described above, while the control group was TAU | Not mentioned | Face to face (group) | Pre-natal clinic | 1 x session | No | No | |
| Ebrahimipour, 2016 | To examine the effectiveness of education on the promotion of oral health in pregnant women based on the Theory of Planned Behaviour | Educational oral health material included lectures, PowerPoint slides, posters, videos, and hands-on-training  A large mouth replica with a toothbrush/floss was used to practice brushing/flossing in the intervention arm  Oral health leaflets were given to the control arm | The intervention arm received an unspecified number of sessions over a two-month period using the material described. The control arm received the leaflet described and no further intervention | Oral and dental health staff employed at the health centre | Face to face (group) | Health centre | Unspecified number of sessions over a two-month period | No | No | |
| George, 2018 | To examine the effectiveness of a Midwifery-Initiated Oral Health Dental Service Program in improving uptake of dental services, oral health knowledge, quality of oral health, oral health status and birth outcomes amongst pregnant women | The Maternal Oral Health Screening Tool was used in the intervention arms to assess the women’s oral health status  Promotional oral health material (unspecified) was given to the control group  Dental assessments (sulcus bleeding, clinical attachment loss and dental caries) | The midwife intervention involved midwives providing oral health education to the pregnant women. The midwives assessed the women’s oral health status using the ‘Maternal Oral Health Screening’ Tool. Dental referrals were offered to women at risk of poor oral health.  The dental intervention included the intervention described above (midwife assessment), additionally, all women (despite their risk of poor oral health) were referred to student dentists for assessment/treatment. At the end of the dental appointments, the women received oral health education, hygiene instructions and dietary counselling.  The control group received the promotional oral health material described.    In the last trimester, alle three arms (regardless of whether they had dental problems) received a final oral assessment by the student dentists. Women with dental issues were referred for treatment after they have given birth | Student dentists and midwives | Face to face (individual) | 3 x large public hospitals in Sydney | 1 x session with the midwife  At least 1 x dental appointment  1 x final oral health assessment conducted by the student dentist | Yes, individual oral health assessment and treatment where needed | No | |
| Ghaffari, 2018 | To examine the effect of an educational intervention based on the Health Belief Model (HBM) in pregnant women | The educational material was delivered using PowerPoint slides and leaflets | The intervention arm received oral health education designed to target the HBM constructs. To target susceptibility, facts about tooth decay was presented. To target perceived severity, images of oral health problems were used. To target barriers, the high cost of dental treatment was addressed in group discussions. Basic dental anatomy was also presented to the women. The content of the sessions was based on credible scientific sources and field specialist’s comments.  The control group was TAU | The lectures were delivered by a specialist dentist | Face to face (group) | Six health centres in western part of Tehran | 3 x sessions of 90 minutes for one month | No | No | |
| Jeihooni, 2017 | To examine the effectiveness of a health education program based on the Health Belief Model on pregnant women’s dental hygiene behaviours | Lectures with PowerPoint slides about oral health in pregnancy were used as educational material, as well as Q&A’s and group discussions  Teeth and mouth replica was used to demonstrate brushing/flossing  Informative videos on oral health care were used to increase the capability of the women in the intervention arm  Pamphlets and posters were used to increase the practical brushing/flossing skills of the women | The intervention consisted of teaching sessions held every other week using the material described. The education was designed based on the Iranian Ministry of Health and Medical Education department’s recommendations. Routine care was also given to the intervention and the control arm | Health care personnel (unspecified) | Face to face (group) | 2 x health centres in Fasa city | 6 x sessions lasting for 1 hour every other week | No | No | |
| Ramazani, 2014 | To examine the effect of two methods of delivering oral health guidance on pregnant women’s oral health knowledge, attitudes, and behaviour, and to determine the effects on clinical gingival status | PowerPoint slides about oral health were used as educational material for the ‘direct group’. Topics including oral health in pregnancy and its impact on pregnancy outcomes, the impact of nutritional deficiencies during pregnancy on oral infant health, and recommendations about oral hygiene practices formed part of the education  Pamphlets with the same information described above were given to the ‘indirect’ group  Oral hygiene toolkits were provided to all women post-test | The intervention consisted of a 20-minute lecture for the women in the ‘direct’ group using the material described  The women in the ‘indirect’ group were given enough time to read the pamphlet and were given the pamphlets after the post-test questionnaire.  Both intervention groups received oral hygiene toolkits.    The control was TAU | Not mentioned | Face to face (group) | Health centre in Zahedan | 1 x session for 20 minutes | No | No | |
| Riedy, 2015 | To examine a behavioral intervention to increase dental attendance among rural Oregonian low-income women and their children | Leaflet from the National Maternal Child Oral Health Resource Centre (NMCOH) that described tips to maternal and child oral health were provided to the women in the pre-natal and post-natal Motivational Interviewing (MI) arm, and the pre-and post-natal health education arms  Gift cards, a baby gift, and toothbrushes and toothpaste for the mother and children were given to the participants to encourage participation  Postcards to ‘check in’ with the women were given in all arms  Follow-up phone calls to ‘check in’ with the women (in all arms)  Fillable written plans for the women to take home after the session were provided for all the women (across arms)  A brief video about parenting behaviours to prevent tooth decay were shown to the women in the post-natal MI arm and the post-natal health education arm  15-minute video about oral were shown to the women in the pre-natal health education arm only | The intervention consisted of MI conducted either in pre (arm 1) or post-natal (arm 2) women.  In the pre-natal MI arm, the counselling was delivered face to face by the counselor immediately after enrolment. The counselors focused on barriers to care during pregnancy and identified the women’s dental health-related needs and engaged with active problem-solving with the women. The women were given the leaflet described in the materials and received information about dental coverage and guidelines for oral health. The counsellors made a follow-up phone call four and six weeks later.  The women in the post-natal MI arm received their counselling face to face around nine months after they gave birth (~10 % received it over the phone). The counselors identified the women’s needs for their child’s dental health-related needs and barriers to professional care and personal oral hygiene. The women were also presented with education on dietary practices for their children and were shown a brief video on parenting behaviours for prevention tooth decay. The content of the video was discussed afterwards. The women also received the same leaflet and follow-up phone call as the women in the pre-natal MI arm.  The health education intervention was also delivered in the pre- or post-natal.  The women in the pre-natal health education arm watched a 15-minute video about oral health in pregnancy and were shown a slideshow with the content of the leaflet from the NMCOH.  The women in the post-natal health education arm watched the same video as the post-natal MI arm, but no discussion of the material followed. | College educated social or health service professionals 2 x counsellors at one site | Face to face (group and individual) | Public health departments | 1 x MI session  1 x health education session  Phone calls (unspecified) | Yes, individual MI counselling sessions | Yes, video recordings and protocols with scripted communication to ensure fidelity | |
| Saffari 2020 | To examine the effectiveness of Motivational Interviewing (MI) as a behavior-change technique to enhance self-efficacy and oral health among pregnant women |  | 30-minute introductory session in groups of 10-12 were held. The goals of the intervention were explained, and a timetable was presented. No oral health education was provided in this session.  MI counselling sessions , where the aim was to establish rapport between the counsellor and participant, provide oral health education, identify barriers/ facilitators to oral health behaviour and identify how to overcome barriers/facilitators were held.  The control arm consisted of oral health education lectures | 1 x expert health educator certified in MI and oral health  1 x oral health technician (control group) | Face to face (group based introductory session)  Face to face MI sessions (individual) | Maternal health clinic in Tehran | 1 x 30-minute introductory session (MI)  2 x 45 minutes over one month (MI)  2 x 1-hour sessions over two weeks (control) | Yes, individual MI counselling sessions | No | |
| Selvarajan, 2019 | To examine pregnant women’s oral health knowledge and attitudes and to test the effectiveness of an oral health education tool | Audiovisual aids with oral health education | The dental health education was given to all women using audio-visual aids and carried out in groups of 20 participants. The presentation was interactive, and the women were allowed to ask question and ask for clarifications where needed | Not mentioned | Face to face (group based) | Government primary health center | 1 x 15 minute | No | No | |
| Shanazi, 2014 | To examine the effect of educational intervention on perceived susceptibility, self-efficacy, and DMFT (Dental Caries Index) of pregnant women | Oral health educational pamphlets were given to the women to ensure educational continuity | The intervention arm was given oral health educational training in four sessions, held in groups of 22 women.  The control group received common education at the health centre | Not mentioned | Face to face (group based) | Health centre in Delfan city | 4 x 90-minute sessions (intervention) | No | No | |
| Nickbin-Poshtamsary, 2018 | To examine the effect of an educational program on dental plaque and caring performance of pregnant mothers in the city of Rasht in Iran | Educational pamphlets and booklets were given to the intervention arm as part of the teaching and formed part of the indirect learning. The materials were based on educational goals from the Ministry of Health and Medical Education | The educational needs of the intervention participants were determined by the questionnaires collected at baseline. Analysis showed that perceived benefits and awareness were the strongest predictors of dental plaque and were therefore used to promote oral health. The educational program consisted of group discussions, lectures, and Q&As.  The control group was TAU | Not mentioned | Face to face (group) | Health centre in Rasht | Not mentioned | No | No | |

**Table 3b**

*Intervention description of the studies with type 2 diabetes participants using the TIDieR Checklist*

| First author, year | Why | What (materials) | What (procedures) | Who provided | How | Where | When and how much | Tailoring (individual) | Fidelity |
| --- | --- | --- | --- | --- | --- | --- | --- | --- | --- |
| Cinar, 2014 | To examine if a health coaching approach compared with formal health education resulted in better outcomes among people with type 2 diabetes (T2D) | Dental assessment for all participants (caries, Community Periodontal Index, and Clinical Attachment Loss)  An informative pamphlet sent out to eligible participants  Letter of welcoming detailing information about the coaching sessions was given to the HC arm participants  Logbook with details of the participant’s coach was provided for the HC participants. The logbook was also used so the participant could track his/her progress  Educational pamphlet about oral health and diabetes was posted to the participants in the HE arm after the training sessions to support learning | The intervention had two arms, the health coach arm (intervention) and the health education arm (active control).  The HC concept was introduced in at the first session and focused on building rapport between the coach. This session also focused on goal setting, an assessment of beliefs and creating action plans. After the first in person session, the participant was coached over the phone after ~14 days. The second session focused on changing beliefs, experiencing, and learning. Patient adherence to the agreed action plan was discussed. Depending on the participant’s progress, further in person sessions were introduced. This session was also follow-up by a coaching session over the phone. The fourth in person session focused on behaviour change and was also followed up by a phone call. The fifth session was focused on behavioural maintenance and self-monitoring and was conducted in person and followed up by a phone call.  The health education arm (active control) received formal education focused on oral health and its relationship with diabetes and quality of life (QoL). The education was delivered as seminars and addressed topics including diabetes, blood glucose monitoring, physical activity, diet, weight loss, medication, smoking cessation, and late complications of diabetes.  All participants across arms were invited for free periodontal cleaning during the intervention period | 2 x dentists with professional International Coaching Council (ICC) training  2 x hygienists with professional ICC training  Professional health coaches  Community dentistry professionals  1 x diabetes specialist nurse  1 x diabetes physician  1 x diabetes dietician | Face to face  Over the phone  (Individual HC arm)  Face to face (group) (control)  Over the phone (individual) (control) | Outpatient clinics from two hospitals in Istanbul | 5-6 x in person individual coaching session  4-5 x 20-60 minutes coaching sessions delivered over the phone  2 x face to face session (control)  4 x phone calls (control) | Yes, individual coaching sessions | No |
| Cinar, 2018 | To assess the effectiveness of health coaching versus health education using clinical subjective measures among people with T2D | See Cinar et al., 2014 | See Cinar et al., 2014 | See Cinar et al., 2014 | See Cinar et al., 2014 | Outpatient clinics from two hospitals in Turkey and Denmark | 5-6 x in person individual coaching session  4-5 x 20-60 minutes coaching sessions delivered over the phone  2 x face to face session (control)  4 x phone calls (control) | Yes, individual coaching sessions | No |
| Hsu, 2021 | To examine the effects of nonsurgical periodontal therapy and community health workers engagement on oral self-care behaviours, periodontal status, and oral health related QoL in patients with T2D | Lecture slides were used to present the educational material on tablets in the intervention arm. The tablets contained a camera function to help participants check their dental cleaning conditions after using a plaque staining agent  Oral health supplies were given to the intervention arm participants (toothbrush, toothpaste, floss, interdental brush, mouthwash cup and mouthwash)  A simple leaflet detailing the relationship between diabetes and oral health was given to the control arm after the periodontal therapy | The intervention arm received non-surgical periodontal therapy and periodontal care education. The dates and times were arranged according to the participants availability. The primary goal of the lessons was to provide information on toothbrush selection tools, correct brushing technique and details about the two-way relationship between diabetes and oral health.  The control group only received non-surgical periodontal therapy and a simple leaflet (see materials). Routine oral, individual health advice was also provided | Community health workers | Face to face (individual) | Metabolism divisions of Kaohsiung Medical University Hospital | 1x session of non-surgical periodontal therapy (intervention and control)  4 x 30-minute sessions on oral health education (intervention) | Yes, individually tailored periodontal therapy and oral health advice | Yes, working log to record the intervention |
| Malekmahmoodi, 2020 | To examine the effect of training based on the Health Belief Model on oral hygiene-related behaviours in people with T2D | The educational material used in the intervention arm was delivered through PowerPoint slides, Q&As, leaflets and booklets  Oral health supplies (toothbrush, toothpaste, and dental floss) | The intervention arm received training based on the Health Belief Model constructs (perceived susceptibility, severity, self-efficacy, barriers, benefits, cues to action and performance of oral health behaviours).  The first session was aimed at increasing the knowledge of diabetes and oral health complications. The second session was targeting susceptibility and severity by presenting statistics of oral health problem prevalence in diabetes. The third session was focused on the perceived benefits, barriers, and cues to action of oral health. The fourth session was focused on self-efficacy and performance. Self-efficacy was targeted by empowering the ability to undertake oral health behaviours by providing the oral health supplies detailed in the materials, and by encouraging the participants to practice brushing/flossing.  The control arm received routine care which included a monthly visit by a doctor, public health educators, a dietician, and a nurse | 1 x doctor  Public health educators  1 x dietician  1 x nurse | Face to face (group) (intervention)  Face to face (individual) (control) | Diabetes clinic in Kashan | 4 x 120-minute sessions for 1 month (intervention)  1 x monthly visit for 30-minutes (control) | No | No |
| Phetnin, 2020 | To examine the effectiveness of the Diabetic and Oral Care Program for Seniors (DOCS) in improving oral health perceptions, behaviours, oral hygiene and reduced glycemic status in older patients with T2D | Lecture PowerPoint slides were used as part of the educational material in the intervention arm. The slides were developed by a general dentist, periodontist, physician, nurse practitioner, and a nutritionist  Oral health supplies were given to the intervention arm (toothbrush, toothpaste, and interdental brushes) | The intervention arm received four weeks of the oral health care program which was based on the Health Belief Model. The first session was focused on oral health and diabetes education. The second session included practicing oral health behaviours and self-examinations. Individual hygiene instructions were also given to the participants along with oral health supplies detailed in the materials  The control arm was TAU and included the participants seeing a doctor one per month | 2 x health officers  2 x dentists | Face to face (group and individual) | Two health centres | 2 x 1-hour sessions | Yes, individual oral hygiene instructions | Not detailed |
| Saengtipbovorn, 2015 | To examine the effectiveness of a Lifestyle, Change plus Dental Care (LCDC) program by improved knowledge, attitude, and practice toward oral health and diabetes among the elderly with T2D | See Saengtipbovorn et al., 2014 | See Saengtipbovorn et al., 2014 | 2 x nurse practitioners trained in Motivational Interviewing (MI) and lifestyle change and dental care  2 x dental assistants trained in MI and lifestyle change and dental care | Face to face (group and individual) | Two health centres in Bangkok | 20-minute educational sessions spread over three months (weekly frequency not detailed)  MI sessions spread over three months (weekly frequency not detailed) | Yes, individual MI sessions and individual lifestyle and oral health instructions | The facilitators were trained in delivery, but no system was in place to assess adherence/fidelity |
| Saengtipbovorn, 2014 | To examine the effectiveness of a Lifestyle, Change plus Dental Care (LCDC) program to improve glycemic and periodontal status in the elderly with type 2 diabetes | A self-regulation manual was given to the intervention arm where participants could identify behavioural goals  The educational material was delivered via PowerPoint slides  Oral health supplies were given to the intervention arm participants (toothbrush, toothpaste, cleaning dentures, and dental floss)  A 15-minute video was shown to the intervention arm participants after the first and second session to reinforce the educational material | In the first session, the intervention arm received one 20-minute lifestyle and oral health and diabetes education. This was followed by individual MI counselling, introduction to the self-regulation manual, and identified behavioral goals relating to oral health and diabetes. The participants also received individual oral hygiene instructions and oral health supplies. All materials outlines were developed in a focus group discussion with ideas from doctors, nurse practitioners, dentists, dental assistants, and a representative for diabetes patients. The materials outlined were also validated by dental, diabetes, and education experts. The control group received TAU | 2 x nurse practitioners trained in MI and lifestyle change and dental care  2 x dental assistants trained in MI and lifestyle change and dental care | Face to face (group and individual) | Two health centres in Bangkok | 20-minute educational sessions spread over three months (weekly frequency not detailed)  MI sessions spread over three months (weekly frequency not detailed) | Yes, individual MI sessions and individual lifestyle and oral health instructions | The facilitators were trained in delivery, but no system was in place to assess adherence/fidelity |

**Table 4a**

*Table 4a maps the intervention components onto the COM-B Model (pregnant population)*

| Reference | Theory | Psychological capability | Physical capability | Social opportunity | Physical opportunity | Reflective motivation | Automatic motivation |
| --- | --- | --- | --- | --- | --- | --- | --- |
| Adams, 2017 | No theory | Oral health education | Toothbrushing/flossing training |  | Provision of oral health supplies |  |  |
| Al Khamis, 2017 | Social Cognitive Theory | Oral health education |  |  | Provision of oral health supplies | Planning of oral health behaviour |  |
| Bahri, 2015 | No theory | Oral health education |  |  |  |  |  |
| Bansal, 2019 | No theory | Oral health education |  |  |  |  |  |
| Cardenas, 2010 | No theory | Oral health education |  |  |  |  |  |
| Chawla, 2017 | No theory | Oral health education |  |  |  |  |  |
| Cilbulka, 2011 | No theory | Oral health education |  |  | Provision of oral health supplies  Providing opportunity for dental appointment and treatment |  |  |
| Clifford, 2012 | No theory | Oral health education |  |  | Provision of oral health supplies |  |  |
| Ebrahimipour, 2016 | Theory of Planned Behaviour | Oral health education | Toothbrushing/flossing training |  |  |  |  |
| George, 2018 | Reinforcement | Oral health education |  |  | Providing opportunity for dental appointment and treatment |  |  |
| Ghaffari, 2018 | The Health Belief Model | Oral health education |  |  |  |  |  |
| Jeihooni, 2017 | The Health Belief Model | Oral health education | Toothbrushing/flossing training |  |  |  |  |
| Ramazani, 2014 | No theory | Oral health education |  |  | Provision of oral health supplies |  |  |
| Riedy, 2015 | Motivational Interviewing | Oral health education |  |  |  |  |  |
| Saffari, 2020 | Motivational Interviewing | Oral health education |  |  |  |  |  |
| Selvarajan, 2017 | No theory | Oral health education |  |  |  |  |  |
| Shanazi, 2014 | The Health Belief Model | Oral health education |  |  |  |  |  |
| Nickbinposhtamsary, 2018 | The Health Belief Model | Oral health education |  |  |  |  |  |

**Table 4b**

*Table 4b maps the intervention components onto the COM-B Model (type 2 diabetes population)*

| First author, year | Theory | COM-B Model | | | | | |
| --- | --- | --- | --- | --- | --- | --- | --- |
|  |  | Capability | | Opportunity | | Motivation | |
|  |  | Psychological | Physical | Social | Physical | Reflective | Automatic |
| Cinar, 2014 | Motivational Interviewing  Self-efficacy  Neurolinguistic Programming | Oral health education |  |  | Providing opportunity for dental appointment and treatment | The intervention targeted self-monitoring |  |
| Cinar, 2018 | Motivational Interviewing  Self-efficacy  Neurolinguistic Programming | Oral health education |  |  | Providing opportunity for dental appointment and treatment | The intervention targeted self-monitoring |  |
| Hsu, 2021 | No theory | Oral health education | Toothbrushing/flossing training |  | Providing opportunity for dental appointment and treatment |  |  |
| Malemahmoodi, 2020 | The Health Belief Model | Oral health education |  |  | Provision of oral health supplies |  |  |
| Phetin, 2020 | The Health Belief Model | Oral health education |  |  | Provision of oral health supplies |  |  |
| Saengtipbovorn, 2014 | Motivational Interviewing  Health Belief Model  Social Cognitive Theory  Cognitive Behavioural Theory | Oral health education |  |  | Provision of oral health supplies |  |  |
| Saengtipbovorn, 2015 | Motivational Interviewing  Health Belief Model  Social Cognitive Theory  Cognitive Behavioural Theory | Oral health education |  |  | Provision of oral health supplies |  |  |

**Reference list of included studies**

1. Adams SH, Gregorich SE, Rising SS, Hutchison M, Chung LH. Integrating a Nurse‐Midwife‐Led Oral Health Intervention Into CenteringPregnancy Prenatal Care: Results of a Pilot Study. J Midwifery Womens Health. 2017 Jul 7;62(4):463–9.

2. al Khamis S, Asimakopoulou K, Newton T, Daly B. The effect of dental health education on pregnant women’s adherence with toothbrushing and flossing - A randomized control trial. Community Dent Oral Epidemiol. 2017 Oct;45(5):469–77.

3. Bahri N, Tohidinik HR, Bahri N, Iliati HR, Moshki M, Darabi F. Educational intervention to improve oral health beliefs and behaviors during pregnancy. Journal of the Egyptian Public Health Association. 2015 Jun;90(2):41–5.

4. Bansal K, Kharbanda O, Sharma J, Sood M, Priya H, Kriplani A. Effectiveness of an integrated perinatal oral health assessment and promotion program on the knowledge in Indian pregnant women. Journal of Indian Society of Pedodontics and Preventive Dentistry. 2019;37(4):383.

5. Cardenas LM, Ross DD. Effects of an oral health education program for pregnant women. J Tenn Dent Assoc. 2010;90(2):23–6; quiz 26–7.

6. Chawla RM, Mitra P, Shetiya SH, Agarwal DR, Narayana DS, Bomble NA. Knowledge, Attitude, and Practice of Pregnant Women regarding Oral Health Status and Treatment Needs following Oral Health Education in Pune District of Maharashtra: A Longitudinal Hospital-based Study. J Contemp Dent Pract. 2017 May;18(5):371–7.

7. Cibulka NJ, Forney S, Goodwin K, Lazaroff P, Sarabia R. Improving oral health in low-income pregnant women with a nurse practitioner-directed oral care program. J Am Acad Nurse Pract. 2011 May;23(5):249–57.

8. Clifford H, Johnson NW, Brown C, Battistutta D. When can oral health education begin? Relative effectiveness of three oral health education strategies starting pre-partum. Community Dent Health. 2012 Jun;29(2):162–7.

9. Ebrahimipour S, Ebrahimipoiur H, Alibakhshian F, Mohamadzadeh M. Effect of education based on the theory of planned behavior on adoption of oral health behaviors of pregnant women referred to health centers of Birjand in 2016. J Int Soc Prev Community Dent. 2016;6(6):584.

10. George A, Dahlen HG, Blinkhorn A, Ajwani S, Bhole S, Ellis S, et al. Evaluation of a midwifery initiated oral health-dental service program to improve oral health and birth outcomes for pregnant women: A multi-centre randomised controlled trial. Int J Nurs Stud. 2018 Jun;82:49–57.

11. Ghaffari M, Rakhshanderou S, Safari-Moradabadi A, Torabi S. Oral and dental health care during pregnancy: Evaluating a theory-driven intervention. Oral Dis. 2018 Nov;24(8):1606–14.

12. Jeihooni A, Jamshidi H, Kashfi S, Avand A, Khiyali Z. The effect of health education program based on health belief model on oral health behaviors in pregnant women of Fasa city, Fars province, south of Iran. J Int Soc Prev Community Dent. 2017;7(6):336.

13. Ramazani N, Zareban I, Ahmadi R, ZadSirjan S, Daryaeian M. Effect of Anticipatory Guidance Presentation Methods on the Knowledge and Attitude of Pregnant Women Relative to Maternal, Infant and Toddler’s Oral Health Care. J Dent (Tehran). 2014 Jan;11(1):22–30.

14. Rawat R, Aswal GS, Dwivedi D. Effect of Oral Health Education (OHE) Interventions on Knowledge, Attitude, Performance towards Oral Health and Oral Hygiene Status among Pregnant Women: A Systematic Review. Journal of Cardiovascular Disease Research . 2020;11(4):373–83.

15. Riedy CA, Weinstein P, Mancl L, Garson G, Huebner CE, Milgrom P, et al. Dental attendance among low-income women and their children following a brief motivational counseling intervention: A community randomized trial. Soc Sci Med. 2015 Nov;144:9–18.

16. Saffari M, Sanaeinasab H, Mobini M, Sepandi M, Rashidi‐Jahan H, Sehlo MG, et al. Effect of a health‐education program using motivational interviewing on oral health behavior and self‐efficacy in pregnant women: a randomized controlled trial. Eur J Oral Sci. 2020 Aug 3;128(4):308–16.

17. Selvarajan N, Krishnan R, Kumar S. Effect of dental health education on the knowledge and attitude among expectant mothers: A questionnaire study. J Pharm Bioallied Sci. 2019;11(6):194.

18. Shahnazi H, Hosseintalaei M, Esteki Ghashghaei F, Charkazi A, Yahyavi Y, Sharifirad G. Effect of Educational Intervention on Perceived Susceptibility Self-Efficacy and DMFT of Pregnant Women. Iran Red Crescent Med J. 2016 Apr 30;18(5).

19. Nickbin Poshtamsary S, Emami Sigaroudi A, Farmanbar R, Radafshar G, Atrkar Roushan Z, Bayat-Movahed S. An Investigation into the Predictors of Behavior Promoting Oral and Dental Health in Pregnant Women Based on the Health Belief Model (HBM). Journal of Dentomaxillofacial Radiology, Pathology and Surgery. 2016 Dec 1;5(3):24–30.

20. Vamos CA, Thompson EL, Avendano M, Daley EM, Quinonez RB, Boggess K. Oral health promotion interventions during pregnancy: a systematic review. Community Dent Oral Epidemiol. 2015 Oct;43(5):385–96.

21. Cinar AB, Oktay I, Schou L. “Smile healthy to your diabetes”: health coaching-based intervention for oral health and diabetes management. Clin Oral Investig. 2014 Sep 21;18(7):1793–801.

22. Cinar AB, Freeman R, Schou L. A new complementary approach for oral health and diabetes management: health coaching. Int Dent J. 2018 Feb;68(1):54–64.

23. Hsu YJ, Chen YH, Lin KD, Lee MY, Lee YL, Yu CK, et al. Clinical Outcomes and Oral Health-Related Quality of Life after Periodontal Treatment with Community Health Worker Strategy in Patients with Type 2 Diabetes: A Randomized Controlled Study. Int J Environ Res Public Health. 2021 Aug 7;18(16):8371.

24. Malekmahmoodi M, Shamsi M, Roozbahani N, Moradzadeh R. A randomized controlled trial of an educational intervention to promote oral and dental health of patients with type 2 diabetes mellitus. BMC Public Health [Internet]. 2020 Mar 4 [cited 2021 Nov 24];20(1). Available from: /pmc/articles/PMC7057556/

25. Abstracts of the 16th International E-Congress of the European Geriatric Medicine Society. Eur Geriatr Med. 2020 Dec 21;11(S1):1–309.

26. Saengtipbovorn S, Taneepanichskul S. Effectiveness of Lifestyle Change Plus Dental Care Program in Improving Glycemic and Periodontal Status in Aging Patients with Diabetes: A Cluster, Randomized, Controlled Trial. J Periodontol. 2015 Apr;86(4):507–15.

27. Saengtipbovorn S, Taneepanichskul S. Effectiveness of lifestyle change plus dental care (LCDC) program on improving glycemic and periodontal status in the elderly with type 2 diabetes. BMC Oral Health. 2014 Dec 16;14(1):72.

28. Ucheka PI, Cinar AB, Ling J, Derek R. A systematic review of the use of common behavioural interventions in oral health and diabetes management. Orapuh Journal. 2021;2(2):e819.
