# Supplementary material for: Psychologically informed oral health interventions in pregnancy and type 2 diabetes: A scoping review
Source: Front Oral Health. 2022 Dec 21;3:1068905. doi: 10.3389/froh.2022.1068905 (PMC9811123; doi:10.3389/froh.2022.1068905)
Supplement: Supplementary file 2 [file Table2.docx]

**Appendices of Search Strategies**

**Appendix A: The search strategy for The Ovid Interface**

Information sources: EMBASE (1947-2022), MEDLINER (1946-2022), HMIC Health Management Information Consortium (1979-2022), Maternity & Infant Care Database (MIDIRS) (1971-2022), APA PsycInfo (1806-2022), Social Policy and Practice (1985-2022), and The Allied and Complimentary Medicine (1985-2022).

1. oral health.mp. [mp=ti, ab, hw, tn, ot, dm, mf, dv, kf, fx, dq, nm, ox, px, rx, an, ui, sy, bt, id, cc, tx, sh, ct, tc, tm, pt]
2. periodont*.mp. [mp=ti, ab, hw, tn, ot, dm, mf, dv, kf, fx, dq, nm, ox, px, rx, an, ui, sy, bt, id, cc, tx, sh, ct, tc, tm, pt]
3. dental health.mp. [mp=ti, ab, hw, tn, ot, dm, mf, dv, kf, fx, dq, nm, ox, px, rx, an, ui, sy, bt, id, cc, tx, sh, ct, tc, tm, pt]
4. oral hygiene.mp. [mp=ti, ab, hw, tn, ot, dm, mf, dv, kf, fx, dq, nm, ox, px, rx, an, ui, sy, bt, id, cc, tx, sh, ct, tc, tm, pt]
5. (instruct* or advice or advise or educat* or tech* or train*).mp. [mp=ti, ab, hw, tn, ot, dm, mf, dv, kf, fx, dq, nm, ox, px, rx, an, ui, sy, bt, id, cc, tx, sh, ct, tc, tm, pt]
6. (oral and intervent*).mp. [mp=ti, ab, hw, tn, ot, dm, mf, dv, kf, fx, dq, nm, ox, px, rx, an, ui, sy, bt, id, cc, tx, sh, ct, tc, tm, pt]
7. (oral and health promotion).mp. [mp=ti, ab, hw, tn, ot, dm, mf, dv, kf, fx, dq, nm, ox, px, rx, an, ui, sy, bt, id, cc, tx, sh, ct, tc, tm, pt]
8. (oral health and behavio?).mp. [mp=ti, ab, hw, tn, ot, dm, mf, dv, kf, fx, dq, nm, ox, px, rx, an, ui, sy, bt, id, cc, tx, sh, ct, tc, tm, pt]
9. pregnan*.mp. [mp=ti, ab, hw, tn, ot, dm, mf, dv, kf, fx, dq, nm, ox, px, rx, an, ui, sy, bt, id, cc, tx, sh, ct, tc, tm, pt]
10. (expect* and mother*).mp. [mp=ti, ab, hw, tn, ot, dm, mf, dv, kf, fx, dq, nm, ox, px, rx, an, ui, sy, bt, id, cc, tx, sh, ct, tc, tm, pt]
11. diabetes mellitus.mp. [mp=ti, ab, hw, tn, ot, dm, mf, dv, kf, fx, dq, nm, ox, px, rx, an, ui, sy, bt, id, cc, tx, sh, ct, tc, tm, pt]
12. type 2 diabetes.mp. [mp=ti, ab, hw, tn, ot, dm, mf, dv, kf, fx, dq, nm, ox, px, rx, an, ui, sy, bt, id, cc, tx, sh, ct, tc, tm, pt]
13. 1 or 2 or 3 or 4
14. 5 or 6 or 7 or 8

15 9 or 10

16 11 or 12

1. 13 and 14 and 15
2. 13 and 14 and 16
3. 13 and 14 and 15 and 16
4. oral health.mh.
5. (periodont* or gingivitis).mh.
6. dental health.mh.
7. oral hygiene.mh.
8. (instruct* or advice or advise or educat* or tech* or train*).mh.
9. (oral and intervent*).mh.
10. (oral health and promotion).mh.
11. (oral health and behavio?).mh.
12. "pregnan*".mh.
13. (expect* and mother*).mh.
14. diabetes mellitus.mh.
15. type 2 diabetes.mh.

32 20 or 21 or 22 or 23

33 24 or 25 or 26 or 27

34 28 or 29

35 30 or 31

1. 32 and 33 and 34
2. 32 and 33 and 35
3. 32 and 33 and 34 and 35
4. 13 and 32

40 14 and 33

41 15 and 34

42 16 and 35

1. 39 and 40 and 41
2. 39 and 40 and 42
3. 39 and 40 and 41 and 42

**Appendix B: The search strategy for CINAHL (1985-2022)**

| S26 | S22 AND S23 AND S24 | Expanders - Apply equivalent subjects Search modes - Boolean/Phrase | Interface - EBSCOhost Research Databases Search Screen - Advanced Search Database - CINAHL | 36 |
| --- | --- | --- | --- | --- |
| S25 | S21 AND S23 AND S24 | Expanders - Apply equivalent subjects Search modes - Boolean/Phrase | Interface - EBSCOhost Research Databases Search Screen - Advanced Search Database - CINAHL | 106 |
| S24 | S16 OR S17 OR S18 OR S19 OR S20 | Expanders - Apply equivalent subjects Search modes - Boolean/Phrase | Interface - EBSCOhost Research Databases Search Screen - Advanced Search Database - CINAHL | Display |
| S23 | S14 OR S15 | Expanders - Apply equivalent subjects Search modes - Boolean/Phrase | Interface - EBSCOhost Research Databases Search Screen - Advanced Search Database - CINAHL | Display |
| S22 | (MM "Diabetes Mellitus, Type 2") OR (MM "Diabetes Mellitus, Gestational") OR "diabetes OR diabetes mellitus OR type 2 diabetes" OR (MM "Pregnancy in Diabetes") | Expanders - Apply equivalent subjects Search modes - Boolean/Phrase | Interface - EBSCOhost Research Databases Search Screen - Advanced Search Database - CINAHL | Display |
| S21 | (MM "Expectant Mothers") OR "pregnant women" OR (MH "Women's Health") OR (MH "Pregnancy Outcomes") OR (MH "Attitude to Pregnancy") OR (MM "Diabetes Mellitus, Gestational") | Expanders - Apply equivalent subjects Search modes - Boolean/Phrase | Interface - EBSCOhost Research Databases Search Screen - Advanced Search Database - CINAHL | Display |
| S20 | (MM "Health Seeking Behaviors (NANDA)") OR (MM "Health Behavior") OR (MM "Knowledge: Health Behaviors (Iowa NOC)") OR (MM "Oral Health Promotion (Iowa NIC)") OR (MM "Health Seeking Behavior Alteration (Saba CCC)") OR (MM "Health Behavior Component (Saba CCC)") OR "oral AND health behavio#r" | Expanders - Apply equivalent subjects Search modes - Boolean/Phrase | Interface - EBSCOhost Research Databases Search Screen - Advanced Search Database - CINAHL | Display |
| S19 | (MH "Psychosocial Intervention") OR (MH "Self-Care: Oral Hygiene (Iowa NOC)") OR (MH "Oral Hygiene") | Expanders - Apply equivalent subjects Search modes - Boolean/Phrase | Interface - EBSCOhost Research Databases Search Screen - Advanced Search Database - CINAHL | Display |
| S18 | (MH "Outcomes of Education") OR (MM "Patient Education") OR "intervention OR advice OR education" OR (MM "Health Education") OR (MH "Psychology, Educational") | Expanders - Apply equivalent subjects Search modes - Boolean/Phrase | Interface - EBSCOhost Research Databases Search Screen - Advanced Search Database - CINAHL | Display |
| S17 | (MM "Oral Hygiene") OR (MM "Self-Care: Oral Hygiene (Iowa NOC)") | Expanders - Apply equivalent subjects Search modes - Boolean/Phrase | Interface - EBSCOhost Research Databases Search Screen - Advanced Search Database - CINAHL | Display |
| S16 | (MM "Dental Health Education") | Expanders - Apply equivalent subjects Search modes - Boolean/Phrase | Interface - EBSCOhost Research Databases Search Screen - Advanced Search Database - CINAHL | Display |
| S15 | (MM "Gingival Diseases") OR (MM "Periodontal Diseases") OR (MM "Periodontal Examination") OR "periodontal disease OR gingivitis" OR (MH "Gingival Recession") OR (MH "Psychosocial Aspects of Illness") | Expanders - Apply equivalent subjects Search modes - Boolean/Phrase | Interface - EBSCOhost Research Databases Search Screen - Advanced Search Database - CINAHL | Display |
| S14 | (MM "Oral Health") OR "oral health" OR (MM "Oral Health Promotion (Iowa NIC)") OR (MM "Health Services Needs and Demand") | Expanders - Apply equivalent subjects Search modes - Boolean/Phrase | Interface - EBSCOhost Research Databases Search Screen - Advanced Search Database - CINAHL | Display |
| S13 | S9 AND S10 AND S11 | Expanders - Apply equivalent subjects Search modes - Boolean/Phrase | Interface - EBSCOhost Research Databases Search Screen - Advanced Search Database - CINAHL | 36 |
| S12 | S8 AND S10 AND S11 | Expanders - Apply equivalent subjects Search modes - Boolean/Phrase | Interface - EBSCOhost Research Databases Search Screen - Advanced Search Database - CINAHL | 106 |
| S11 | S3 OR S4 OR S5 OR S6 OR S7 | Expanders - Apply equivalent subjects Search modes - Boolean/Phrase | Interface - EBSCOhost Research Databases Search Screen - Advanced Search Database - CINAHL | 91,613 |
| S10 | S1 OR S2 | Expanders - Apply equivalent subjects Search modes - Boolean/Phrase | Interface - EBSCOhost Research Databases Search Screen - Advanced Search Database - CINAHL | 43,678 |
| S9 | (MM "Diabetes Mellitus, Type 2") OR (MM "Diabetes Mellitus, Gestational") OR "diabetes OR diabetes mellitus OR type 2 diabetes" OR (MM "Pregnancy in Diabetes") | Expanders - Apply equivalent subjects Search modes - Boolean/Phrase | Interface - EBSCOhost Research Databases Search Screen - Advanced Search Database - CINAHL | 59,760 |
| S8 | (MM "Expectant Mothers") OR "pregnant women" OR (MH "Women's Health") OR (MH "Pregnancy Outcomes") OR (MH "Attitude to Pregnancy") OR (MM "Diabetes Mellitus, Gestational") | Expanders - Apply equivalent subjects Search modes - Boolean/Phrase | Interface - EBSCOhost Research Databases Search Screen - Advanced Search Database - CINAHL | 109,929 |
| S7 | (MM "Health Seeking Behaviors (NANDA)") OR (MM "Health Behavior") OR (MM "Knowledge: Health Behaviors (Iowa NOC)") OR (MM "Oral Health Promotion (Iowa NIC)") OR (MM "Health Seeking Behavior Alteration (Saba CCC)") OR (MM "Health Behavior Component (Saba CCC)") OR "oral AND health behavio#r" | Expanders - Apply equivalent subjects Search modes - Boolean/Phrase | Interface - EBSCOhost Research Databases Search Screen - Advanced Search Database - CINAHL | 31,625 |
| S6 | (MH "Psychosocial Intervention") OR (MH "Self-Care: Oral Hygiene (Iowa NOC)") OR (MH "Oral Hygiene") | Expanders - Apply equivalent subjects Search modes - Boolean/Phrase | Interface - EBSCOhost Research Databases Search Screen - Advanced Search Database - CINAHL | 6,497 |
| S5 | (MH "Outcomes of Education") OR (MM "Patient Education") OR "intervention OR advice OR education" OR (MM "Health Education") OR (MH "Psychology, Educational") | Expanders - Apply equivalent subjects Search modes - Boolean/Phrase | Interface - EBSCOhost Research Databases Search Screen - Advanced Search Database - CINAHL | 55,098 |
| S4 | (MM "Oral Hygiene") OR (MM "Self-Care: Oral Hygiene (Iowa NOC)") | Expanders - Apply equivalent subjects Search modes - Boolean/Phrase | Interface - EBSCOhost Research Databases Search Screen - Advanced Search Database - CINAHL | 2,874 |
| S3 | (MM "Dental Health Education") | Expanders - Apply equivalent subjects Search modes - Boolean/Phrase | Interface - EBSCOhost Research Databases Search Screen - Advanced Search Database - CINAHL | 413 |
| S2 | (MM "Gingival Diseases") OR (MM "Periodontal Diseases") OR (MM "Periodontal Examination") OR "periodontal disease OR gingivitis" OR (MH "Gingival Recession") OR (MH "Psychosocial Aspects of Illness") | Expanders - Apply equivalent subjects Search modes - Boolean/Phrase | Interface - EBSCOhost Research Databases Search Screen - Advanced Search Database - CINAHL | 11,628 |
| S1 | (MM "Oral Health") OR "oral health" OR (MM "Oral Health Promotion (Iowa NIC)") OR (MM "Health Services Needs and Demand") | Expanders - Apply equivalent subjects Search modes - Boolean/Phrase | Interface - EBSCOhost Research Databases Search Screen - Advanced Search Database - CINAHL |  |

**Appendix C: The search strategy for the Cochrane Database**

1. Title and abstract keyword: oral health education
2. OR title and abstract keyword: dental health education
3. AND title and abstract keyword: intervention
4. OR title and abstract keyword: health promotion
5. AND title and abstract keyword: pregnancy
6. Title and abstract keyword: oral health education
7. OR title and abstract keyword: dental health education
8. AND title and abstract keyword: intervention
9. OR title and abstract keyword: health promotion
10. AND title and abstract keyword: diabetes
